# Publication proportions for registered breast cancer trials: before and following the introduction of the ClinicalTrials.gov results database

**DOI:** 10.1186/s41073-016-0017-4

**Published:** 2016-07-18

**Authors:** Innocent Gerald Asiimwe, Dickson Rumona

**Affiliations:** 1 University of Liverpool, Liverpool, L69 3BX UK; 2 Liverpool Reviews and Implementation Group (LRiG), 2.06 Whelan Building, The Quadrangle, Brownlow Hill, Liverpool, L69 3GB UK

**Keywords:** Breast cancer, ClinicalTrials.gov, Incomplete publication, Registries, Results databases

## Abstract

**Background:**

To limit selective and incomplete publication of the results of clinical trials, registries including ClinicalTrials.gov were introduced. The ClinicalTrials.gov registry added a results database in 2008 to enable researchers to post the results of their trials as stipulated by the Food and Drug Administration Amendment Act of 2007. This study aimed to determine the direction and magnitude of any change in publication proportions of registered breast cancer trials that occurred since the inception of the ClinicalTrials.gov results database.

**Methods:**

A cross-sectional study design was employed using ClinicalTrials.gov, a publicly available registry/results database as the primary data source. Registry contents under the subcategories ‘Breast Neoplasms’ and ‘Breast Neoplasms, Male’ were downloaded on 1 August 2015. A literature search for included trials was afterwards conducted using MEDLINE and DISCOVER databases to determine publication status of the registered breast cancer trials.

**Results:**

Nearly half (168/340) of the listed trials had been published, with a median time to publication of 24 months (Q1 = 14 months, Q3 = 42 months). Only 86 trials were published within 24 months of completion. There was no significant increase in publication proportions of trials that were completed before the introduction of the results database compared to those completed after (OR = 1.00, 95 % CI = .61 to 1.63; adjusted OR = 0.84, 95 % CI = .51 to 1.39). Characteristics associated with publication included trial type (observational versus interventional adjusted OR = .28, 95 % CI = .10 to .74) and completion/termination status (terminated versus completed adjusted OR = .22, 95 % CI = .09 to .51).

**Conclusions:**

Less than a half of breast cancer trials registered in ClinicalTrials.gov are published in peer-reviewed journals.

**Electronic supplementary material:**

The online version of this article (doi:10.1186/s41073-016-0017-4) contains supplementary material, which is available to authorized users.

## Background

“Full and transparent reporting of trials is crucial to ensure that decisions about health care are based on the best available evidence.” [1]. Selective, incomplete or delayed reporting of trial results (for instance, due to their statistical significance, effect size or direction) distorts the available evidence, compromises systematic reviews, renders evidence-based decisions inaccurate, wastes resources and is unethical [2–10]. There is evidence of selective, incomplete and delayed reporting across several if not all fields, including cancer, cardiovascular disease and neurological disorders [11, 12]; pain [13]; surgery [14]; arthritis [15]; oral health [16]; eczema [17]; and acupuncture [18]. Registration of trials at their inception and appropriate follow-up was proposed to enable the tracking of protocol changes as well as improve research transparency, accountability and integrity [8]. Examples of trial databases/registries established to accomplish these objectives are ClinicalTrials.gov and the European Clinical Trials Database (EudraCT) launched by the US National Institute of Health (NIH) in 2000 and the European Medicines Agency (EMA) in 2004 respectively [19, 20].

Despite the establishment of these registries, the ultimate aim of trial registration (full and transparent reporting) has not been achieved. Empirical evidence demonstrates a continued low compliance to the required reporting standards [8]. For instance, Ross and colleagues [21] reported that less than a half of trials funded by the National Institute of Health (*n* = 635) were published within 30 months of being completed. In addition to the incomplete reporting, there was a lack of transparency with trial outcomes being changed to favour positive or statistically significant findings [22]. These results were consistent to those reported before trial registries were established [23–27].

Earlier studies have reported that investigators are less likely to submit negative or null findings [6, 24]. Originally, trial registries/databases did not mandate the submission of trial findings; however, it was hoped that an additional mandatory requirement to post results of registered trials would improve completeness and transparency in reporting [8]. Once posted, it would be impossible to conceal negative or null findings. Theoretically, removing this reason for not publishing would possibly, among other factors, increase trial publication rates. The Food and Drug Administration Amendments Act of 2007 (FDAAA) [28] paved way for mandatory (specified group of trials) as well as voluntary posting of results to a results database that was added to the ClinicalTrials.gov registry in 2008. Although there are some contradictory reports [29], both trial registration and mandatory results reporting have been shown to improve publication rates [30–32].

Studies have previously assessed delayed or incomplete publication in several specialties (including cancer trials in general) [11, 12, 33, 34], with some assessing the influence of the introduction of the ClinicalTrials.gov results database on publication rates [29, 30, 32]. A search of the literature failed to identify reports related to this issue in relation to breast cancer trials. Considering that breast cancer is one of the leading causes of morbidity and mortality in the world today [35, 36], this study sought to (1) determine the proportion of registered breast cancer trials (i.e. initiated after February 2000) completed/terminated on or before 31 July 2013 that were published in peer-reviewed journals overall and within 24 months of completion; (2) compare publication rates for trials completed before introduction of a results database (from initiation of the database in February 2000 to 31 September 2008) with those completed afterwards (from 1 October 2008 to 31 July 2013); and (3) outline the main trial characteristics associated with results’ publication. ClinicalTrials.gov was used because not only is it the largest publicly available registry (195,624 registered trials as of 30 July 2015) but it also combines the registry with a results database [8, 37].

## Methods

### Study design

This was a cross-sectional study of breast cancer trials registered at ClinicalTrials.gov.

### Search strategy

ClinicalTrials.gov was searched on 1 August 2015 by browsing through the different conditions by category/topic. Under the category ‘Cancers and Other Neoplasms’, the two subcategories ‘Breast Neoplasms’ and ‘Breast Neoplasms, Male,’ were chosen and the registry contents downloaded.

### Selection of relevant trials

#### Inclusion criteria

Both experimental and observational trials under the subcategories ‘Breast Neoplasms’ and ‘Breast Neoplasms, Male,’ with primary completion or termination dates up to 31 July 2013 were included (this was to allow for a 24 month period; 1 August 2013 to 31 July 2015). The primary completion date was defined as the date of collection of the primary outcome measure for the last included patient [38] (when unreported, the expected completion date as defined by the trial investigators was used); whereas termination date as a premature/early date on which participant recruitment, examination and, or treatment stops with the trial not scheduled to start again [38].

#### Exclusion criteria

‘Ongoing’ trials, trials initiated before/during February 2000, trials completed after 31 July 2013, terminated trials that did not enrol a single participant and trials with ‘unknown’ status or unknown completion dates were excluded. ‘Ongoing’ trials were defined as trials that were ‘active, but not recruiting’, ‘suspended’, ‘enrolling by invitation’, ‘recruiting’, ‘not yet recruiting’, ‘available for expanded access’ and ‘temporarily not available for expanded access’, whereas trials with ‘unknown status’ were those trials whose recruitment status had not been verified within the previous two years for ‘recruiting’ or ‘not yet recruiting’ studies [38]. A track of trials included per study period was kept with trials in a given period excluded once an accumulated number of 170 trials in the same period was reached.

#### Size of selected sample

This was based on the ‘sample size calculation formula for a difference in proportions’ [39]. Using the Ramsey and Scoggins [33] publication rate of *p*_1_ = 17.6 % (before the release of the results database), 90 % power and assuming a type I error rate of 5 %, power calculations were undertaken under various publication rate increments including 10 % (724 trials), 15 % (340 trials) and 20 % (199 trials). The 15 % absolute change in publication rate (i.e. *p*_2_ = 32.6 %), which produced 340 trials in total or 170 trials per study period, was decided on since it reflected a reasonably large increment whilst still requiring a manageable sample size.

#### Sampling technique

A web-based random number generator [40] was used to generate a sequence of random numbers from within the range of 1 to 6389, 6389 being the number of breast cancer trials registered with ClinicalTrials.gov as of 1 August 2015 (Table 1). Using the generated sequence, the first 170 trials from each trial period fulfilling inclusion/exclusion criteria (below) were selected.

**Table 1:** Breast cancer trials registered with ClinicalTrials.org as of 1 August 2015

Breast cancer category (number of trials)	ClinicalTrials.org number (study rank)	Allocated survey number
Breast Neoplasms (6109 studies)	1–6109	1–6109
Breast Neoplasms, Male (280 studies)	1–280	6110–6389

### Data extraction

A Microsoft Office Excel template was used to extract information from downloaded content including trial characteristics (design: type, phase, randomization status, control status, blinding, interventional model, and endpoint classification; population: age and gender; sample size; study location; registration before/after initiation; and, primary sponsor); completion/termination status (completed or terminated, month and year of completion/termination, completed before/after 1 October 2008 and registered before/after completion/termination); results’ posting in ClinicalTrials.gov (results posted or not; time to posting results); and trial publication status (results published or not, journal of publication, and time to publication).

### Search for corresponding publications

For the literature search of published trials, the publication link (or citation) within the ClinicalTrials.gov, if available, was used. If no link (or citation) was available, a search was conducted in MEDLINE using the ClinicalTrials.gov identification number (CTN). If no publication was found using this number, another search using the keywords ‘breast cancer’ and the study intervention(s)’ primary outcomes and/or principal investigator (if named) was conducted. Articles were matched to the registry information using the study description, primary and secondary objectives, location, enrolment start and end dates, etc. For multiple publications, the publications that most closely fitted the study description in the registry records were chosen. If a decision could not be made on this basis (i.e. for two or more publications both/all closely fitting the registry records) or, if the same publication was published twice, the earliest publication (including electronic publications) was used. For studies whose publication records were not found using the MEDLINE search, a similar search was conducted using University of Liverpool’s DISCOVER. DISCOVER is the University of Liverpool’s electronic library database which contains/links to sources from more than 489 other databases including MEDLINE, Scopus, Science Citation Index, Science Direct, CINAHL Plus, Cochrane database of systematic reviews, EBSCO, ProQuest, Bandolier, etc. [41]) The search included publications for all languages with non-English publications included if a translated abstract and/or main text was available. For published trials, the journal and date of publication, the database in which published records were found and the study’s visibility using a CTN search as well as the reporting of the CTN in the title/abstract were captured. Unpublished studies that had meeting abstracts were also documented but not included as full publications.

From both the ClinicalTrials.gov and publication records, other measures were derived including registered before/after initiation/completion, registered within 21 days if not prospectively registered, time in months from receipt of certification or request for extension to delay results, time in months from completion to publication/posting in ClinicalTrials.org and whether or not results were posted 12 (or published 24) months or less. Finally and depending on the year of publication, each journals’ impact factor was obtained by viewing the respective ‘Journal Citation Reports’ available from the ISI Web of Science. Data collection was completed on 31 August 2015.

### Data cleaning

This involved the review of trial registries to check for and rectify typographical errors. Missing/unclear/inconsistent data variables were resolved as shown below (completion dates were not altered since they had been used to classify the registry records into the two study periods):

- Some missing data was resolved (imputed) by using other ClinicalTrials.gov information. For instance, since all single-group assignments/designs are non-randomized, open-label and have no control group [38], all studies that had missing information under ‘randomization status’, ‘blinding status’ and/or ‘control group’ were imputed accordingly if they were labelled ‘single-group assignment’. For those that had inconsistent data, e.g. a ‘single-group assignment’ also labelled as ‘placebo-controlled,’ the study’s description in the narrative text, if available, and/or the study’s objectives were used to resolve the issue. Similarly, the study’s objectives were used to impute missing ‘endpoint classifications’ whereas studies with more than one listed site or conducted in more than one country had to be ‘multicentre’.
- For studies with publications, unresolved missing/inconsistent data was imputed using information from published records. For inconsistencies between registry and published records. e.g. achieved sample size, published records took precedence.
- For information especially age ranges that did not perfectly match the data categories as per the data extraction template, the closest category was recorded, e.g. a study recruiting participants ≥21 years but <70 years was considered to be in the ‘Adults (≥18 years, <65 years) only’ category.

Finally, to aid data analysis, all missing/blank fields were completed using a ‘0’ except for those fields that required ‘time to completion, in months’ (in relation to results posting within ClinicalTrials.gov and publication in peer-reviewed journals), ‘sample size’ and ‘number of multiple centres’.

### Analysis

The IBM Statistical Package for Social Sciences (SPSS) Version 21 was used. Descriptive analysis was used to identify the main trial characteristics as well as the proportions of the main outcome measures. Associations between selected variables were examined using chi-square tests (both variables categorical), independent sample *T* test/Mann-Whitney/logistic regression (one categorical, one quantitative) or linear regression (both quantitative). Conducted tests included:

- Tests between the two study periods versus the trial characteristics to ascertain whether/not trial characteristics were different across the two periods
- Tests between the two study periods versus publication status (published/not within 24 months and time to publication) to compare publication rates and timeliness across the two periods
- Tests between publication status (results published/not) versus trial characteristics to determine which trial characteristics were associated with trial publication

The Kaplan-Meier survival method was used to compare the cumulative probability of trials being published within 24 months for the two study periods (all trials without publications after a 24-month follow-up were censored). Finally, all factors that were associated to publication status (*p* value ≤.25) [42] were introduced in a multivariate logistic regression to identify factors independently associated with publication status. To examine the effects of multiple testing, the Bonferroni adjustment method [43] was employed.

## Results

### Trial selection and characteristics

**Fig. 1:**
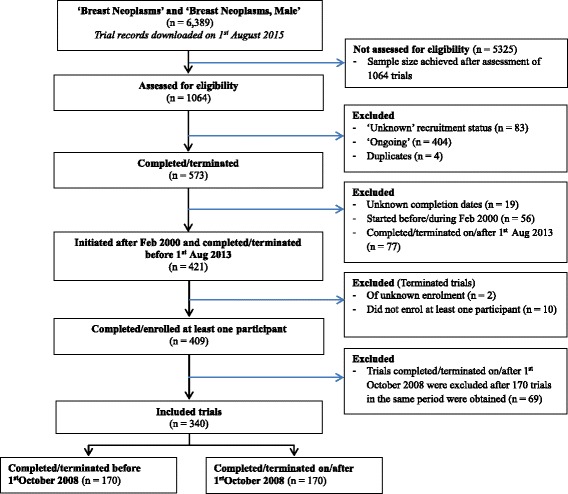
Selection of trials

A total of 340 registered trials were included as shown in Fig. 1 (also see Additional file 1). The majority of these trials were interventional, phase I/II or II, non-randomized, had no control, open-label, of single-group assignment and assessed both safety and efficacy (Table 2). The median sample size was 48 (first quartile, *Q*_1_ = 24, third quartile, *Q*_3_ = 118) with most trials including female participants aged ≥18 years old. Most trials were multicentre; and, of the multicentre trials that reported the number of centres (*n* = 87), the median number of sites was 9 (*Q*_1_ = 4, *Q*_3_ = 23). The primary sponsor was categorized as non-industry/non-government in 60.9 % of the trials. The registration, completion/termination and results posting statuses of the same trials are also presented in Table 2. A notable observation is that most (70.9 %) trials were not prospectively registered (i.e. before initiation). When registration within 21 days of initiation was considered, an extra 34 trials were registered adequately. The median year or trial completion was 2008 (*Q*_1_ = 2007, *Q*_3_ = 2011). Only 59 (17.4 %) trials had posted results with a median time to posting results of 24 months (*Q*_1_ = 13 months, *Q*_3_ = 49 months) (of these, only 14 had posted results within the required 12 months). For trials without posted results, only 7 had applied for a certification or request for extension to delay results. However, these trials had not posted results even after a median time of 31 months (from receipt of the certification/request by ClinicalTrials.gov). The greatest proportions of unknown/missing values were recorded in the categories ‘number of centres’ and ‘number of centres, if multiple’ which respectively had 37.1 and 47.0 % values missing.

**Table 2:** Trial characteristics, comparison between the two study periods and publication status.

Trial characteristics		Frequency^c^	Completion/termination^c^		*p* value^a^	Publication Status^d^		*p* value^a^
			Before 1 Oct 2008	On/after 1 Oct 2008		Published	Not Published	
Type	Interventional	297 (87 %)	158 (93 %)	139 (82 %)	.002	159 (54 %)	138 (47 %)	<.0001
	Observational	43(13 %)	12 (7 %)	31 (18 %)		9 (21 %)	34 (79 %)	
Phase	I	47 (14 %)	28 (21 %)	19 (17 %)	.917^b^	21 (45 %)	26 (55 %)	.161^b^
	I/II or II	145 (43 %)	80 (59 %)	65 (60 %)		85 (59 %)	60 (41 %)	
	II/III or III	45 (13 %)	24 (18 %)	21 (19 %)		29 (64 %)	16 (36 %)	
	IV	8 (2 %)	4 (3 %)	4 (4 %)		6 (75 %)	2 (25 %)	
	Not applicable (observational studies)	43 (13 %)						
	Unknown (missing values)	52 (15 %)						
Randomization status	Randomized	107 (32 %)	60 (39 %)	47 (34 %)	.409	63 (59 %)	44 (41 %)	.229
	Non-randomized	186 (55 %)	95 (61 %)	91 (66 %)		96 (52 %)	90 (48 %)	
	Not applicable (observational studies)	43 (13 %)						
	Unknown (missing values)	4 (1 %)						
Control status	Placebo only	29 (9 %)	16 (10 %)	13 (10 %)	.136^b^	15 (52 %)	14 (48 %)	.519^b^
	Active-control only	74 (22 %)	34 (22 %)	40 (29 %)		41 (55 %)	33 (45 %)	
	Both placebo and active-control	17 (5 %)	13 (8 %)	4 (3 %)		12 (71 %)	5 (29 %)	
	No control	172 (51 %)	92 (59 %)	80 (58 %)		90 (52 %)	82 (48 %)	
	Not applicable (observational studies)	43 (13 %)						
	Unknown (missing values)	5 (2 %)						
Blinding	Open-label	255 (75 %)	128 (82 %)	127 (93 %)	.006^b^	134 (53 %)	121 (48 %)	.286^b^
	Single-blind	9 (3 %)	5 (3 %)	4 (3 %)		4 (44 %)	5 (56 %)	
	Double (or Triple) blind	30 (9 %)	24 (15 %)	6 (4 %)		20 (67 %)	10 (33 %)	
	Not applicable (observational studies)	43 (13 %)						
	Unknown (missing values)	3 (1 %)						
Interventional model	Parallel	107 (32 %)	54 (35 %)	53 (39 %)	.432^b^	60 (56 %)	47 (44 %)	.184^b^
	Crossover	9 (3 %)	7 (5 %)	2 (2 %)		4 (44 %)	5 (56 %)	
	Factorial	4 (1 %)	2 (1 %)	2 (2 %)		4 (100 %)	0 (0 %)	
	Single-group assignment	172 (51 %)	92 (59 %)	80 (58 %)		90 (52 %)	82 (48 %)	
	Not applicable (observational studies)	43 (13 %)						
	Unknown (missing values)	5 (2 %)						
Endpoint classification	Safety only	28 (8 %)	15 (10 %)	13 (10 %)	.336^b^	12 (43 %)	16 (57 %)	.069^b^
	Efficacy only	103 (30 %)	49 (32 %)	54 (40 %)		49 (48 %)	54 (52 %)	
	Both Safety and Efficacy	160 (47 %)	91 (59 %)	69 (51 %)		96 (60 %)	64 (40 %)	
	Not applicable (observational studies)	43 (13 %)						
	Unknown (missing values)	6 (2 %)						
Age	Included adults (≥18 years, >65 years)	51 (15 %)	28 (18 %)	23 (15 %)	.697^b^	29 (57 %)	22 (43 %)	.716^b^
	Included adults and older adults (≥65 years)	237 (70 %)	121 (76 %)	116 (75 %)		117 (49 %)	120 (51 %)	
	Included adults and children (<18 years)	5 (2 %)	2 (1 %)	3 (2 %)		2 (40 %)	3 (60 %)	
	Included older adults (≥65 years) only	20 (6 %)	8 (5 %)	12 (8 %)		9 (45 %)	11 (55 %)	
	Included children (<18 years) only	0 (0 %)	0 (0 %)	0 (0 %)		0 (0 %)	0 (0 %)	
	Unknown (missing values)	27 (8 %)						
Gender	Male only	1 (0 %)	0 (0 %)	1 (1 %)	.136^b^	0 (0 %)	1 (100 %)	.132^b^
	Female only	225 (66 %)	106 (62 %)	119 (70 %)		105 (47 %)	120 (53 %)	
	Both male and female	114 (34 %)	64 (38 %)	50 (29 %)		63 (55 %)	51 (45 %)	
Number of Centres	Single	50 (15 %)	18 (17 %)	32 (29 %)	.035	34 (68 %)	16 (32 %)	1.000
	Multiple	164 (48 %)	87 (83 %)	77 (71 %)		112 (68 %)	52 (32 %)	
	Unknown (missing values)	126 (37 %)						
Number of Countries	Single	301 (89 %)	148 (88 %)	153 (91 %)	.469	143 (48 %)	158 (53 %)	.013
	Multiple	36 (11 %)	20 (12 %)	16 (10 %)		25 (69 %)	11 (31 %)	
	Unknown (missing values)	3 (1 %)						
Country	US/Canada only	209 (62 %)	105 (63 %)	104 (62 %)	.855^b^	100 (48 %)	109 (52 %)	.013^b^
	International (outside USA/Canada) only	102 (30 %)	49 (29 %)	53 (31 %)		48 (47 %)	54 (53 %)	
	USA/Canada and International	26 (8 %)	14 (8 %)	12 (7 %)		20 (77 %)	6 (23 %)	
	Unknown (missing values)	3 (1 %)						
Primary sponsor	Industry	91 (27 %)	45 (27 %)	46 (27 %)	.609	45 (50 %)	46 (51 %)	.545^b^
	Government (US and non-US)	42 (12 %)	24 (14 %)	18 (11 %)		24 (57 %)	18 (43 %)	
	Non-industry/non-government	207 (61 %)	101 (59 %)	106 (62 %)		99 (48 %)	108 (52 %)	
Registered before initiation	Yes	59 (17 %)	22 (14 %)	37 (27 %)	.005	35 (59 %)	24 (41 %)	.171
	No	241 (71 %)	139 (86 %)	102 (73 %)		119 (49 %)	122 (51 %)	
	Unknown (missing dates)	40 (12 %)						
Registered within 21 days of initiation	Yes	93 (27 %)	30 (19 %)	63 (30 %)	.000	47 (51 %)	46 (50 %)	.980
	No	217 (64 %)	127 (81 %)	90 (59 %)		110 (51 %)	107 (49 %)	
	Unknown (missing dates)	30 (9 %)						
Completed/terminated	Completed	288 (85 %)	148 (87 %)	140 (82 %)	.228	155 (54 %)	133 (46 %)	<.0001
	Terminated	52 (15 %)	22 (13 %)	30 (18 %)		13 (25 %)	39 (75 %)	
Registered before completion/termination?	Yes	283 (84 %)	131 (78 %)	152 (90 %)	.002	135 (48 %)	148 (52 %)	.155
	No	55 (16 %)	38 (23 %)	17 (10 %)		32 (58 %)	23 (42 %)	
	Unknown (missing dates)	2 (1 %)						
Results posted in ClinicalTrials.gov?	Yes	59 (17 %)	20 (12 %)	39 (23 %)	.007	31 (53 %)	28 (48 %)	.597
	No	281 (83 %)	150 (88 %)	131 (77 %)		137 (49 %)	144 (51 %)	
Publication link available	Yes	77 (23 %)	41 (24 %)	36 (21 %)	.517	77 (100 %)	0 (0 %)	<.0001
	No	263 (77 %)	129 (76 %)	134 (79 %)		91 (35 %)	172 (51 %)	

^a^Pearson chi-square *p* values used unless otherwise. For cells with expected cell count less than 5, exact *p* values (Fisher’s exact test or Monte Carlo significance) were used

^b^Likelihood ratio *p* value

^c^Percentages by column for frequencies and study period comparisons (some percentages may not add up to 100 % due to rounding)

^d^Percentages by row for publication comparisons

A comparison of trial characteristics between the two study periods for continuous variables using the Mann-Whitney *U* test, i.e. sample size (*N* = 340, *U* = 14,447.0, Sig. =.997), number of multiple centres (*N* = 87, *U* = 915.0, Sig. =.857) and time to posting of results (*N* = 59, *U* = 237.5, Sig. =.014) showed that only the latter was statistically significantly different across the periods. Other comparisons are shown in Table 2. Overall, 8/22 factors/characteristics differed between the two periods including trial type, blinding status, number of centres (single versus multiple), registration before trial initiation, registration within 21 days of trial initiation, registration before completion/termination and result’s posting (including time to posting) in ClinicalTrials.gov.

### Overall publication of study results

**Fig. 2:**
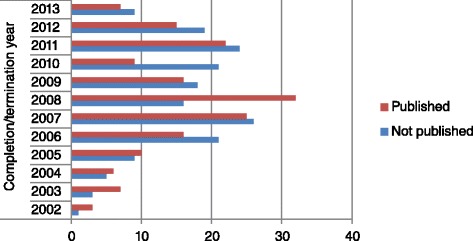
Publication (number of trials) by year of completion/termination

Of the included trials (*n* = 340), only 77 had valid publication links. The links (*n* = 77) and a MEDLINE search using the ‘ClinicalTrials.gov identifier (CTN)’ (*n* = 5) and ‘applicable search terms’ (*n* = 86) produced a total of 168 trials (Additional file 2) published by MEDLINE-indexed journals (with a median impact factor of 4.17, *Q*_1_ = 2.63, *Q*_3_ = 6.41). Apart from 16 meeting abstracts (excluded), the DISCOVER search did not yield any more publications. Therefore, 49.4 % (168/340) of breast cancer trials registered with ClinicalTrials.gov were published, with a median time to publication of 24 months (*Q*_1_ = 14 months, *Q*_3_ = 42 months). The publication trend based on year of completion/termination is shown in Fig. 2. Considering publication timeliness, only 86 (51.2 %) of the 168 published trials (which translates to 25.3 % of the 340 registered trials) were published within 24 months of trial completion. With regard to ‘visibility’, less than half (45.8 %, 77/168), a third (27.4 %, 46/168) and a fifth (14.9 %, 25/168) of published trials had publication links, were obtainable using the CTN search and reported the CTN in the title/abstract, respectively.

### Publication across the two study periods

**Fig. 3:**
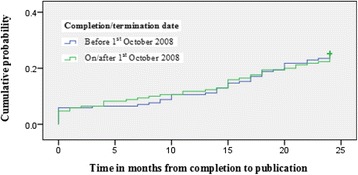
A comparison of the cumulative probability of trials being published over time across the two study periods (all trials followed up for 24 months)

Of the 168 trials published overall, 93 (55.4 %) trials were completed/terminated before 1 October 2008 compared to 75 (44.6 %) that were completed/terminated on/after 1 October 2008. Both trial periods had an equal number of trials (*n* = 43, 25.3 % of 170 trials) being published within 24 months. Chi-square tests did not reveal an association between completion/termination before/after 1 October 2008 and results being published within 24 months/not (OR = 1.00, 95 % CI = .61 to 1.63, *p* value = 1.000, *n* = 340; OR adjusted for type and completion/termination status = 0.84, 95 % CI = .51 to 1.39, *p* value = .493, *n* = 340). Concerning time to publication, Kaplan-Meier survival analysis (Fig. 3) did not find a significant difference between the two time periods (log rank test *p* value = .989).

### Study characteristics associated with trial publication

The associations between publication status (results published/not) with trial characteristics (Table 2) revealed that observational studies were less likely to be published than interventional studies (OR = .23, 95 % CI = .11 to .50, *p* value < .0001, *n* = 340) whereas terminated trials were less likely to be published than those completed as planned (OR = .29, 95 % CI = .15 to .56, *p* value <.0001, *n* = 340). There was also a significant difference in publication rates between trials conducted in single countries and those conducted in multiple countries (OR = .40, 95 % CI = .19 to .84, *p* value = .013, *n* = 337). Finally, the country of location was significantly associated with publication status (*p* value = .013). Although the availability of a publication link is shown in Table 2 (*p* value < .0001), the presence of a publication link was not considered a predisposing factor to publication because of a clear temporal relationship (publication precedes establishment of a link). None of the continuous variables was significantly associated with publication status (Mann-Whitney *U* tests were sample size, *N* = 340, *U* = 12716.0, Sig. =.056; number of multiple centres, *N* = 87, *U* = 275.5, Sig. =.944; and time to posting of results, *N* = 59, *U* = 407.5, Sig. =.687 whereas logistic regression ORs (95 % CIs) were sample size, OR = 1.00, 95 % CI = 1.00 to 1.00; number of multiple centres, OR = .98, 95 % CI = .94 to 1.03; and time to posting of results, OR = 1.00, 95 % CI = .98 to 1.02).

**Fig. 4:**
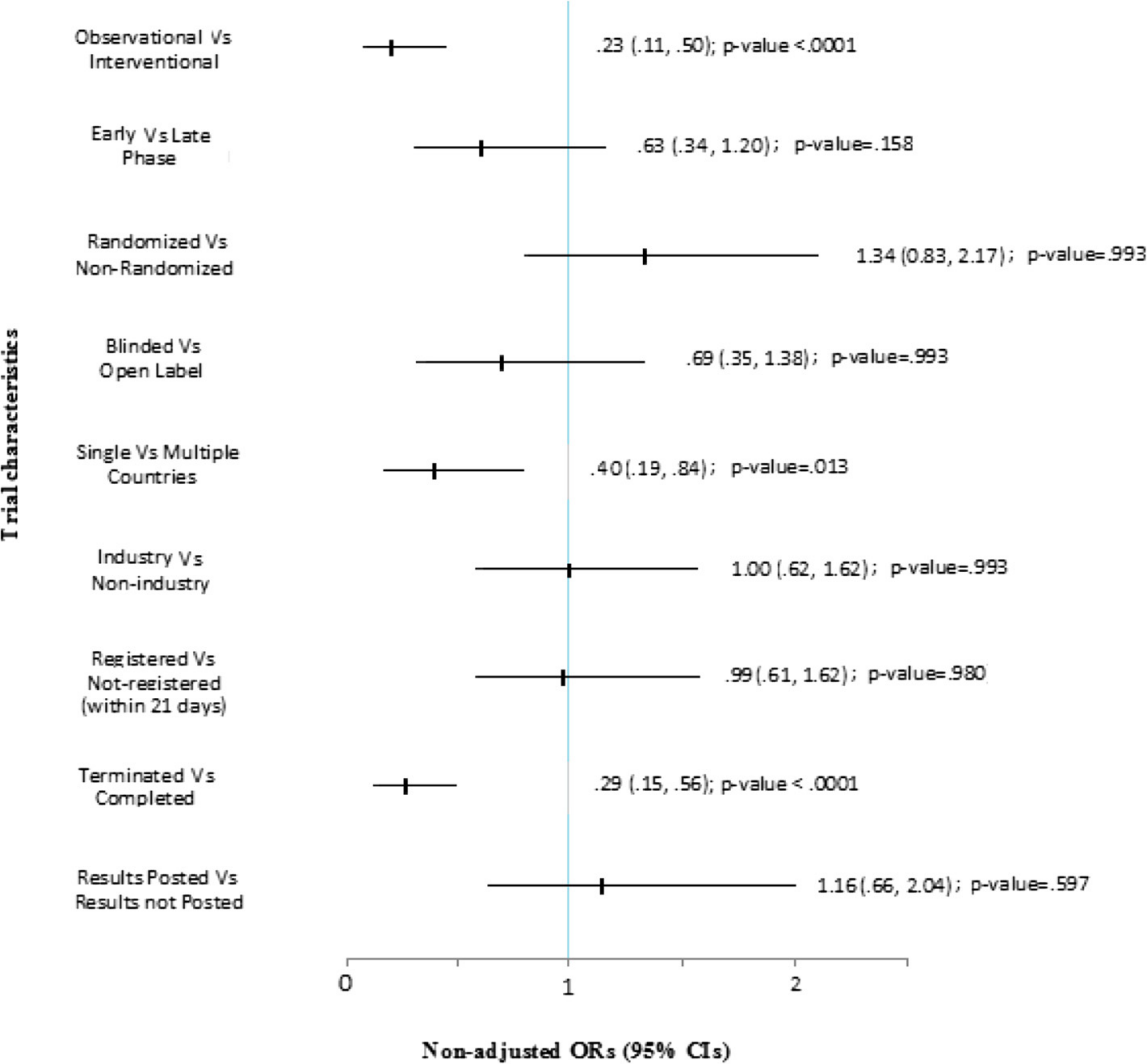
Non-adjusted ORs (95 % CIs) of results publication for selected trial (dichotomous) characteristics

Chi-square comparisons between early phase (phase I and I/II or II) versus late phase (phase II/III or III and IV), blinded (single, double or triple blind) versus not-blinded (open-label) and industry versus non-industry funding with regard to results publication were also compared. The results (together with associations of other dichotomous trial characteristics) are presented in Fig. 4.

On multivariate analysis, only trial type (observational versus interventional studies) (adjusted OR = .28, 95 % CI = .10 to .74) and completion/termination status (terminated versus completed as planned) (adjusted OR = .22, 95 % CI = .09 to .51) were associated with publication status.

### Examination of the effects of multiple testing

A total of 45 tests (21 comparing trial characteristics across the study periods, 2 between the study periods and publication status and 22 examining trial characteristics associated with publication status) were conducted. According to Bland [43], these many tests inflate the *α* (type I) error. For the overall study error to remain at the set α level, the Bonferroni adjustment sets a new error rate of 1 − (1 − *α*)^*n*^ (which approximates *α*/*n*), where *n* is the number of tests performed. 0.05/45 = .001. Using this *p* value, only two associations (results published/not with trial type and results published/not with completion/termination status) remained significant.

## Discussion

### Principal findings

This study was designed to measure the publication proportion for registered breast cancer trials and detect any changes in the same after the 2008 introduction of the ClinicalTrials.gov results database. First and foremost, the proportion of trials published within 24 months was very low at 25.3 % (increasing to 49.4 % when publication timeliness is not considered). Although these figures are higher than those earlier reported (for oncology trials) [11, 33], a majority of registered trials remain unpublished, which is worrisome given that oncology was among the first medical fields to widely adopt trial registration [44]. Overall, more trials were published in the period prior to compared to that after 1 October 2008 (55.4 versus 44.6 %). However, it is not possible to associate this difference to the introduction of the results database since trials conducted before 1 October 2008 had a longer follow-up [45]. Indeed, this difference ceased to exist when all trials were followed up for the same 24-month period (50 versus 50 %).

For both study periods, a majority of studies remained unpublished. This study however did not evaluate the reasons such studies were not published. Reasons for non-publication of research studies have nevertheless been discussed elsewhere [7, 11, 33, 45–48]. It is, however, also possible that some studies designated as ‘unpublished’ were still undergoing preparation for publication or editorial review and will soon be published [49, 50]. This is especially important since the study’s minimum follow-up period was 2 years yet according to Hopewell et al. [50], positive and negative findings are on average published within 4–5 and 6–8 years respectively. Schmucker and colleagues also noted that the probability of publication within 2 years, for their study, ranged from 7 to 30 % [51]. As Manzoli [52] demonstrated publication rates increase with time, with a follow-up of 355 vaccine trials showing publication rates of 12, 29, 53 and 73 % after 12, 24, 36 and 48 months after completion, respectively. For this study therefore, trials completed before 1 October 2008 with a minimum follow-up of 7 years should have had enough time for publication. Worryingly, 45.3 % (77/170) of these trials were not published which is in line with Khan et al.’s [15] report that 20 to 70 % of trials remain unpublished even after a follow-up of several years.

Only trial type (interventional versus observational) and completion/termination status were associated with trial publications. As mentioned earlier, registration of trials was introduced among other reasons to ensure the complete reporting of the same trials [8]. With FDAAA [28] among other laws not mandating their registration, exacerbated by the fact that the medical community has given them little attention with regard to their need to be registered [53], it is no surprise that the publication proportion of observational trials was much less than that of interventional trials. However as Williams and colleagues [53] explain, most, if not all, ethical and scientific reasons that prompted registration of interventional studies also apply to observational studies. They should therefore also be registered (and results publicly disseminated). With regard to completion/termination status, reasons similar/related to those for termination (e.g. recruitment failure, safety concerns, futility, economic reasons, etc.) [31, 54] may discourage those initiating/conducting/funding the same trials from investing more time/money into their publication. However, as Shamliyan and Kane [31] report, reporting of such trials is especially important if they were terminated for safety concerns. Lessons learnt from such trials are important in shaping future research [3].

### Study strengths and weaknesses

To the best of the researchers’ knowledge, this is the first study to determine publication rates in the breast cancer field and compare such rates before and after the introduction of the ClinicalTrials.gov results database. ClinicalTrials.gov is currently the largest registry/results database which contains trials conducted from almost all areas of the world. It is also publicly available meaning any researcher can access and replicate the study if scientifically/ethically justifiable. By allowing a 2-year period, the study ensured that completed/terminated trials had at least 2 years to be published in peer-reviewed journals. However, several limitations are recognized.

- First, the study was designed to detect a 15 % change in publication rate yet the observed change was 10.6 % (54.7 to 44.1 %). A difference of 10 % required a sample size of 724 trials. The study’s smaller sample size produced less precise results (wider CIs) which could have affected the statistical significance of some of the associations as well as their interpretation [42].
- Secondly, although ClinicalTrials.gov is the largest publicly available database, the majority of trials originate from the USA/Canada (Table 2) which as Herrmann et al. [55] note might limit generalizability of the findings.
- Thirdly, the study relied on the accuracy of information in the ClinicalTrials.gov registry. However, this has previously been found to be unreliable [52, 56]. Incomplete, inconsistent or inaccurate records (e.g. some included studies were published before their registered completion dates) negatively impact the validity of the study’s findings. For instance, as Ross et al. report, studies providing completion dates are generally more likely to be published than those not providing the same dates [7]. This implies that this study, by not including studies without completion dates, may have overestimated the proportion of published studies in the registry. Additionally, because studies did not provide the date/day of completion, the time to publication (months) was approximate. Where it was possible to rectify inconsistencies/inaccurate/incomplete information, this was done as described under the ‘Data cleaning’ section.
- Fourthly, although the Bonferroni adjustment was used to assess the effects of multiple testing, it was not employed to adjust for the type I error. The Bonferroni adjustment discourages multiple tests yet these are necessary for the interpretation of findings; it also increases the type II error (‘false negatives’) [57]. As Perneger [57] summarizes, “…simply describing what tests of significance have been performed, and why, is generally the best way of dealing with multiple comparisons.” The number of tests conducted (and why) were described in the analysis section. It should be noted that the ‘primary’ outcome (proportion of published trials) which was in the first instance not significant would not be affected by any Bonferroni adjustment. The only affected tests were ‘secondary’ (i.e. used to interpret the ‘primary’ finding) and were therefore of less relevance to the overall effect of multiple testing on the study’s results.
- Fifthly, the study design did not include the assessment of unpublished findings. Nevertheless, the registry does not contain contact information for completed trials (contains only for ‘recruiting’ or ‘not yet recruiting’ trials) [58] which would have made it difficult had the researcher opted to contact investigators to assess unpublished findings.
- Sixthly, it is possible that the study missed some published reports. However, as Manzoli states, any publications missed using the study’s systematic search are in essence not ‘publicly available’ or ‘visible’ to the public and are unlikely to be identified during subsequent/routine searches [52].
- Finally, a single researcher derived information from the ClinicalTrials.gov registry in addition to searching for publication records. The lack of a second independent search/data extraction lessens scientific vigour since it is impossible to rectify (and/or quantify) issues identifiable thorough researcher disagreements [49].

### Policy implications

This study has several implications. First, the lack of publications or delays in publishing trial results means that at any one time, available evidence will be incomplete [2–10, 59]. Incomplete evidence in turn affects most, if not all, populations including researchers (conducting systematic reviews or further research that needs to be guided by current evidence), health service providers (making treatment decisions), policy makers (making treatment guidelines), patients with their friends and families (final healthcare consumers) and healthcare funders who pay for the treatment interventions. Wrong policy decisions, treatments given or research conducted is wasteful of limited resources. Secondly, patients, healthcare providers, funders, ethical boards, etc. participate in/fund or approve research with the hope that results will be disseminated and used to inform clinical practice. A failure to publish research findings violates these agreements/understandings and is not only unethical but could be regarded as scientific misconduct. In the long run, it erodes the public trust in clinical research [2–10, 59]. It is hoped that all concerned (including but not limited to researchers, journal editors, peer reviewers, sponsors, policymakers, regulators, Institutional Review Boards or Research Ethics Committees, etc.) will continue to work toward the attainment of complete and transparent reporting in the breast cancer and other fields (the OPEN (Overcome failure to Publish nEgative fiNdings)) project has developed targeted recommendations to that effect [60]) so that ultimately patients, healthcare professionals, funders, researchers, etc. benefit through ethical, scientific and efficient utilization and advancement of medical knowledge.

## Conclusions

Less than a half of breast cancer trials registered in ClinicalTrials.gov are published in peer-reviewed journals. Although this is an overall improvement from earlier reported oncology trials [11, 33], the results of the findings suggests the results of the majority of breast cancer trials remain unavailable to the public. These findings raise both ethical and scientific concerns and question both the completeness and validity of the evidence base that guides treatment decisions/guidelines, further research among others with regard to breast cancer treatments. Additionally, they question the effectiveness of the measures including registries and results databases (or their implementation) that have over time been introduced to limit selective, incomplete and delayed publication.

### Recommendations

As Zarin and Tse explain with regard to trial registration, “…the infrastructure…is in place, but culture change by all stakeholders…is necessary before key goals can be reached.” [61] It is important therefore that this ‘cultural change’ be facilitated and the existing infrastructure strengthened. This among others includes increasing the capability of ClinicalTrials.gov to detect and enable rectification of incomplete, inaccurate or inconsistent entries (especially applicable optional data elements) (see Table 2 for proportions of incomplete entries). Responsible authorities should be made accountable for complete, accurate and up-to-date information. ClinicalTrials.gov should retain contact information for completed (especially unpublished) trials. The trial registry should also include a field that enables viewers determine whether/not a study is required to post results as mandated by FDAAA. There should be enforcements (rewards and punishments) at all levels; e.g. Institutional Review Boards, peer reviewers or journal editors should not respectively approve, approve for publication or publish (unless otherwise/justifiable) applicable trials that are not prospectively registered, do not have trial registration numbers or have not posted results as required by the FDAAA (many published trials did not fulfil these requirements).

## Abbreviations

CONSORT, Consolidated Standards of Reporting Trials; EMA, European Medicines Agency; EudraCT, European Clinical Trials Database; FDAAA, Food and Drug Administration Amendment Act of 2007; NIH, US National Institute of Health; OPEN, Overcome failure to Publish nEgative fiNdings; WHO, World Health Organization

## Additional files

Additional file 1: Data extraction template. File containing the 340 registered trials included in the study with the extracted data. (XLSX 115 kb)

Additional file 2: References for identified published reports. References for the 168 trials identified as published by MEDLINE-indexed journals. (DOCX 61 kb)
