# Supplementary material for: Publication proportions for registered breast cancer trials: before and following the introduction of the ClinicalTrials.gov results database
Source: Res Integr Peer Rev. 2016 Jul 18;1:10. doi: 10.1186/s41073-016-0017-4 (PMC5803577; doi:10.1186/s41073-016-0017-4)
Supplement: Supplementary file 2 — References for identified published reports. References for the 168 trials identified as published by MEDLINE-indexed journals. (DOCX 61 kb) [file 41073_2016_17_MOESM2_ESM.docx]

**Additional File 2** References for identified published reports

| **S/N** | **ClinicalTrials.org Identification # (NCT)** | **Search strategy/terms** | **Publication** |
| --- | --- | --- | --- |
| 1 | NCT00434356 | Sunitinib AND Bevacizumab AND Paclitaxel AND Breast Cancer | Mayer, E.L. et al (2010) ‘SABRE-B: an evaluation of paclitaxel and bevacizumab with or without sunitinib as first-line treatment of metastatic breast cancer,’ *Ann Oncol*.; 21(12):2370-6. doi: 10.1093/annonc/mdq260. PMID: 20497961 |
| 2 | NCT00005822 | SU5416 AND Doxorubicin AND Breast Cancer AND Overmoyer B | Overmoyer, B. et al. (2007) ‘Inflammatory breast cancer as a model disease to study tumor angiogenesis: results of a phase IB trial of combination SU5416 and doxorubicin’, *Clin Cancer Res*.; 13(19):5862-8 PMID: 17908980 |
| 3 | NCT00096057 | NCT | Pegram, M.D. et al. (2009) ‘Phase I dose escalation pharmacokinetic assessment of intravenous humanized anti-MUC1 antibody AS1402 in patients with advanced breast cancer’, *Breast Cancer* Res.; 11(5):R73. doi: 10.1186/bcr2409 PMID: 19811637 |
| 4 | NCT00574301 | ClinicalTrials.gov Publication Link | Klimberg, V.S. et al. (2011) ‘Feasibility of percutaneous excision followed by ablation for local control in breast cancer’, *Ann SurgOncol*.; 18(11):3079-87. doi: 10.1245/s10434-011-2002-y. Epub 2011 Sep 9. PMID: 21904959 |
| 5 | NCT01849133 | NCT | Veronesi, U. et al. (2013) ‘Intraoperative radiotherapy versus external radiotherapy for early breast cancer (ELIOT): a randomised controlled equivalence trial’, *Lancet Oncol.*; 14(13):1269-77. doi: 10.1016/S1470-2045(13)70497-2. Epub 2013 Nov 11. PMID: 24225155 |
| 6 | NCT01009008 | Link | Connell, T.F. (2014) ‘Patient-activated controlled expansion for breast reconstruction using controlled carbon dioxide inflation: confirmation of a feasibility study’, *PlastReconstr Surg*.; 134(4):503e-11e. doi: 10.1097/PRS.0000000000000551. PMID: 25357043 |
| 7 | NCT00852930 | Low Level Laser Treatment AND Breast Cancer Related Lymphedema AND Ridner S | Ridner, S.H. et al. (2013) ‘A pilot randomized trial evaluating low-level laser therapy as an alternative treatment to manual lymphatic drainage for breast cancer-related lymphedema’, *OncolNurs Forum*.; 40(4):383-93. doi: 10.1188/13.ONF.383-393. PMID: 23803270 |
| 8 | NCT00766532 | Link | Tevaarwerk, A. et al. (2012) ‘Aromatase inhibitors and calcium absorption in early stage breast cancer’, *Breast Cancer Res Treat*.; 134(1):245-51. doi: 10.1007/s10549-012-1982-z. Epub 2012 Feb 18. PMID: 22350731 |
| 9 | NCT00680667 | Trametes Versicolor AND Breast Cancer AND Torkelson C | Torkelson, C.J. et al. (2012) ‘Phase 1 Clinical Trial of Trametes versicolor in Women with Breast Cancer’, *ISRN Oncol.*;2012:251632. doi: 10.5402/2012/251632. Epub 2012 May 30. PMID: 22701186 |
| 10 | NCT01587248 | Link | Khan, S., Khan, S., Chawla, T. &Murtaza, G. (2014) ‘Harmonic scalpel versus electrocautery dissection in modified radical mastectomy: a randomized controlled trial,’ *Ann SurgOncol*.; 21(3):808-14. PMID: 24232511 |
| 11 | NCT00120016 | Link | Djuric, Z. et al. (2008) ‘Design of a Mediterranean exchange list diet implemented by telephone counseling,’ *J Am Diet Assoc*.; 108(12):2059-65. doi: 10.1016/j.jada.2008.09.006. PMID: 19027409 |
| 12 | NCT00802945 | Link | Awada, A. et al. (2013) ‘NKTR-102 Study Group. Two schedules of etirinotecanpegol (NKTR-102) in patients with previously treated metastatic breast cancer: a randomised phase 2 study,’ *Lancet Oncol*.; 14(12):1216-25. doi: 10.1016/S1470-2045(13)70429-7. Epub 2013 Oct 4. PMID: 24095299 |
| 13 | NCT00365599 | Vorinostat and Tamoxifen and Breast Cancer and Minton S | Munster, P.N. et al. (2011) ‘A phase II study of the histone deacetylase inhibitor vorinostat combined with tamoxifen for the treatment of patients with hormone therapy-resistant breast cancer’, *Br J Cancer*.; 104(12):1828-35. doi: 10.1038/bjc.2011.156. Epub 2011 May 10. PMID: 21559012 |
| 14 | NCT00077376 | Trastuzumab and Ixabepilone (BMS-247550) and Carboplatin and Breast Cancer and Moulder S | Moulder, S. et al. (2010) ‘A phase II trial of trastuzumab plus weekly ixabepilone and carboplatin in patients with HER2-positive metastatic breast cancer: an Eastern Cooperative Oncology Group Trial,’ *Breast Cancer Res Treat*.; 119(3):663-71. doi: 10.1007/s10549-009-0658-9. PMID: 20012354 |
| 15 | NCT00528567 | NCT | Cameron, D. et al. (2013) ‘Adjuvant bevacizumab-containing therapy in triple-negative breast cancer (BEATRICE): primary results of a randomised, phase 3 trial’, *Lancet Oncol*.;14 (10):933-42. doi: 10.1016/S1470-2045(13)70335-8. Epub 2013 Aug 7. PMID: 23932548 |
| 16 | NCT01203267 | Link | Chen, X.S. et al. (2010) ‘Weekly paclitaxel plus carboplatin is an effective nonanthracycline-containing regimen as neoadjuvant chemotherapy for breast cancer’, *Ann Oncol*.; 21(5):961-7. doi: 10.1093/annonc/mdq041. Epub 2010 Mar 8. PMID: 20211870 |
| 17 | NCT00050141 | Link | Johnston, S.R. et al. (2008) ‘A phase II, randomized, blinded study of the farnesyltransferase inhibitor tipifarnib combined with letrozole in the treatment of advanced breast cancer after antiestrogen therapy,’ *Breast Cancer Res Treat*.; 110(2):327-35. Epub 2007 Sep 13. PMID: 17851757 |
| 18 | NCT01147965 | Link | Morse, M.A. et al. (2013) ‘Novel adenoviral vector induces T-cell responses despite anti-adenoviral neutralizing antibodies in colorectal cancer patients,’ *Cancer ImmunolImmunother*.; 62(8):1293-301. doi: 10.1007/s00262-013-1400-3. Epub 2013 Apr 30. PMID: 23624851 |
| 19 | NCT01173497 | INIPARIB AND triple negative breast cancer AND Brain Metastasis | Anders, C. et al. (2014) ‘TBCRC 018: phase II study of iniparib in combination with irinotecan to treat progressive triple negative breast cancer brain metastases’, *Breast Cancer Res Treat*.; 146(3):557-66. doi: 10.1007/s10549-014-3039-y. Epub 2014 Jul 8. PMID: 25001612 |
| 20 | NCT00135018 | Neoadjuvant Chemotherapy AND Celecoxib AND Invasive Breast Cancer AND Chow L | Chow, L.W. et al. (2013) ‘Concurrent celecoxib with 5-fluorouracil/epirubicin/cyclophosphamide followed by docetaxel for stages II - III invasive breast cancer: the OOTR-N001 study’, *Expert OpinInvestig Drugs*.; 22(3):299-307. doi: 10.1517/13543784.2013.766715. PMID: 23394482 |
| 21 | NCT02115984 | Link | Proskurina, A.S. et al. (2015) ‘Results of multicenter double-blind placebo-controlled phase II clinical trial of Panagen preparation to evaluate its leukostimulatory activity and formation of the adaptive immune response in patients with stage II-IV breast cancer,’ *BMC Cancer*.; 15:122. doi: 10.1186/s12885-015-1142-z. PMID: 25886605 |
| 22 | NCT00957112 | Link | Molassiotis, A. et al. (2013) ‘A randomized, controlled trial of acupuncture self-needling as maintenance therapy for cancer-related fatigue after therapist-delivered acupuncture,’ *Ann Oncol*.; 24(6):1645-52. doi: 10.1093/annonc/mdt034. Epub 2013 Feb 21. PMID: 23436910 |
| 23 | NCT00595062 | Quadrant high-dose intraoperative radiation therapy AND breast cancer AND McCormick B | Sacchini, V. et al. (2008) ‘Study of quadrant high-dose intraoperative radiation therapy for early-stage breast cancer’, *Br J Surg*.; 95(9):1105-10. doi: 10.1002/bjs.6208. PMID: 18690634 |
| 24 | NCT00087152 | Link | Chew, H.K. et al. (2008) ‘A phase II study of imatinibmesylate and capecitabine in metastatic breast cancer: Southwest Oncology Group Study 0338’, *Clin Breast Cancer*.;8(6):511-5. doi: 10.3816/CBC.2008.n.062. PMID: 19073506 |
| 25 | NCT00522262 | Link | Woolcott, C.G. et al. (2010) ‘Mammographic density change with 1 year of aerobic exercise among postmenopausal women: a randomized controlled trial,’ *Cancer Epidemiol BiomarkersPrev*.; 19(4):1112-21. doi: 10.1158/1055-9965.EPI-09-0801. Epub 2010 Mar 23. PMID: 20332266 |
| 26 | NCT00938652 | Link | O'Shaughnessy, J. et al. (2014) ‘Phase III study of iniparib plus gemcitabine and carboplatin versus gemcitabine and carboplatin in patients with metastatic triple-negative breast cancer,’ *J ClinOncol*.; 32(34):3840-7. doi: 10.1200/JCO.2014.55.2984. Epub 2014 Oct 27. PMID: 25349301 |
| 27 | NCT00540800 | Link | Nuzzo, F. et al. (2011) ‘Effects on quality of life of weekly docetaxel-based chemotherapy in patients with locally advanced or metastatic breast cancer: results of a single-centre randomized phase 3 trial,’ *BMC Cancer*.; 11:75. doi: 10.1186/1471-2407-11-75. PMID: 21324184 |
| 28 | NCT00172068 | Link | Banys, M. et al. (2013) ‘Influence of zoledronic acid on disseminated tumor cells in bone marrow and survival: results of a prospective clinical trial,’ *BMC Cancer*.; 13:480. doi: 10.1186/1471-2407-13-480. PMID: 24128322 |
| 29 | NCT00705315 | Link | Tryfonidis, K. et al. (2013) ‘A multicenter phase I-II study of docetaxel plus epirubicin plus bevacizumab as first-line treatment in women with HER2-negative metastatic breast cancer,’ *Breast*.; 22(6):1171-7. doi: 10.1016/j.breast.2013.08.017. Epub 2013 Oct 1. PMID: 24091128 |
| 30 | NCT01329627 | Metronomic Chemotherapy AND Paclitaxel AND Doxorubicin AND Cyclophosphamide AND Breast Cancer | Petry, V. et al. (2015) ‘Metronomic chemotherapy in the neoadjuvant setting: results of two parallel feasibility trials (TraQme and TAME) in patients with HER2+ and HER2- locally advanced breast cancer,’ *Braz J Med Biol Res*.; 48(5):479-85. doi: 10.1590/1414-431X20144354. Epub 2015 Mar 6. PMID: 25760024 |
| 31 | NCT00547989 | Paravertebral Block AND General Anaesthesia for Breast Surgery AND Bouman E | Bouman, E.A. et al. (2014) ‘Continuous paravertebral block for postoperative pain compared to general anaesthesia and wound infiltration for major oncological breast surgery’, *Springerplus*.; 3:517. doi: 10.1186/2193-1801-3-517. eCollection 2014. PMID: 25279309 |
| 32 | NCT00532272 | Goserelin or Leuprorelin AND Letrozole AND Breast Cancer AND Ro J | Park, I.H. et al. (2010) ‘Phase II parallel group study showing comparable efficacy between premenopausal metastatic breast cancer patients treated with letrozole plus goserelin and postmenopausal patients treated with letrozole alone as first-line hormone therapy’, *J ClinOncol*.; 28(16):2705-11. doi: 10.1200/JCO.2009.26.5884. Epub 2010 Apr 26. PMID: 20421538 |
| 33 | NCT00795678 | NCT | Morikawa, A. et al. (2015) ‘Capecitabine and lapatinib uptake in surgically resected brain metastases from metastatic breast cancer patients: a prospective study’, *Neuro Oncol*.; 17(2):289-95. doi: 10.1093/neuonc/nou141. Epub 2014 Jul 11. PMID: 25015089 |
| 34 | NCT00450866 | Epothilone B OR Patupilone AND Brain OR CNS Metastases AND Breast Cancer AND Peereboom D | Peereboom, D.M. et al. (2014) ‘Phase II trial of patupilone in patients with brain metastases from breast cancer’, *Neuro Oncol*.; 16(4):579-83. doi: 10.1093/neuonc/not305. Epub 2014 Jan 26. PMID: 24470546 |
| 35 | NCT00689975 | Diet and Exercise AND Breast Cancer AND Saxton JM | Scott E. et al. (2013) ‘Effects of an exercise and hypocaloric healthy eating program on biomarkers associated with long-term prognosis after early-stage breast cancer: a randomized controlled trial’, *Cancer Causes Control*.; 24(1):181-91. doi: 10.1007/s10552-012-0104-x. Epub 2012 Nov 27. PMID: 23184120 |
| 36 | NCT00088998 | Link | Perez, E.A. et al. (2010) ‘North Central Cancer Treatment Group (NCCTG) N0432: phase II trial of docetaxel with capecitabine and bevacizumab as first-line chemotherapy for patients with metastatic breast cancer,’ *Ann Oncol*.; 21(2):269-74. doi: 10.1093/annonc/mdp512. Epub 2009 Nov 9. PMID: 19901014 |
| 37 | NCT01038804 | YM155 AND Docetaxel AND Breast Cancer | Clemens, M.R. (2015) ‘Phase II, multicenter, open-label, randomized study of YM155 plus docetaxel as first-line treatment in patients with HER2-negative metastatic breast cancer,’ *Breast Cancer Res Treat*.; 149(1):171-9. doi: 10.1007/s10549-014-3238-6. Epub 2014 Dec 30. PMID: 25547219 |
| 38 | NCT00017095 | Link | Bonnefoi, H. et al. (2011) ‘TP53 status for prediction of sensitivity to taxane versus non-taxane neoadjuvant chemotherapy in breast cancer (EORTC 10994/BIG 1-00): a randomised phase 3 trial’, *Lancet Oncol*.; 12(6):527-39. doi: 10.1016/S1470-2045(11)70094-8. Epub 2011 May 11. PMID: 21570352 |
| 39 | NCT01458457 | Link | Witt, C.M. et al. (2015) ‘Effectiveness of an additional individualized multi-component complementary medicine treatment on health-related quality of life in breast cancer patients: a pragmatic randomized trial,’ *Breast Cancer Res Treat*.; 149(2):449-60. doi: 10.1007/s10549-014-3249-3. Epub 2015 Jan 3. PMID: 25555830 |
| 40 | NCT00525161 | Link | Massarweh, S. et al. (2014) ‘Impact of adding the multikinase inhibitor sorafenib to endocrine therapy in metastatic estrogen receptor-positive breast cancer,’ *Future Oncol*.; 10(15):2435-48. doi: 10.2217/fon.14.99. PMID: 24826798 |
| 41 | NCT00721058 | Delineation AND Tumorbed AND Breast Cancer AND Boersma LJ | Boersma, L.J. (2011) ‘Is contrast enhancement required to visualize a known breast tumor in a pre-operative CT scan?’ *RadiotherOncol*.; 100(2):271-5. doi: 10.1016/j.radonc.2011.06.027. Epub 2011 Jul 7. PMID: 21741717 |
| 42 | NCT00622674 | Bortezomib and Cetuximab AND Solid Tumors Expressing EGFR AND Dudek A | Dudek, A.Z. et al. (2009) ‘Phase I study of bortezomib and cetuximab in patients with solid tumours expressing epidermal growth factor receptor,’ *Br J Cancer*; 100(9):1379-84. doi: 10.1038/sj.bjc.6605043. PMID: 19401697 |
| 43 | NCT00393172 | Link | Smith, A.J. et al. (2013) ‘The effects of aerobic exercise on estrogen metabolism in healthy premenopausal women,’ *Cancer Epidemiol Biomarkers Prev*.; 22(5):756-64. doi: 10.1158/1055-9965.EPI-12-1325. PMID: 23652373 |
| 44 | NCT00087399 | Link | Loprinzi, C.L. et al. (2007) ‘Phase III trial of gabapentin alone or in conjunction with an antidepressant in the management of hot flashes in women who have inadequate control with an antidepressant alone: NCCTG N03C5,’ *J ClinOncol*.; 25(3):308-12. Epub 2006 Dec 4. PMID: 17146104 |
| 45 | NCT00028639 | Single Agent PS-341 (Bortezomib) AND Breast Cancer AND Gradishar W | Engel, R.H. et al. (2007) ‘A phase II study of single agent bortezomib in patients with metastatic breast cancer: a single institution experience,’ *Cancer Invest*.; 25(8):733-7. Epub 2007 Oct 18. PMID: 17952740 |
| 46 | NCT00917748 | Link | Hovey, E. et al. (2014) ‘Phase III, randomized, double-blind, placebo-controlled study of modafinil for fatigue in patients treated with docetaxel-based chemotherapy,’ *Support Care Cancer*.; 22(5):1233-42. doi: 10.1007/s00520-013-2076-0. Epub 2013 Dec 17. PMID: 24337761 |
| 47 | NCT00060320 | Link | Pockaj, B.A. et al. (2006) ‘Phase III double-blind, randomized, placebo-controlled crossover trial of black cohosh in the management of hot flashes: NCCTG Trial N01CC1,’ *J ClinOncol*.; 24(18):2836-41. PMID: 16782922 |
| 48 | NCT00900835 | Pain and Fatigue Management in Patients With Breast Cancer AND Ferrell B | Borneman, T. et al. (2010) ‘Reducing patient barriers to pain and fatigue management,’ *J Pain Symptom Manage*.; 39(3):486-501. doi: 10.1016/j.jpainsymman.2009.08.007. PMID: 20303026 |
| 49 | NCT00051051 | CI-1033 AND metastatic breast cancer | Rixe, O. et al. (2009) ‘A randomized, phase II, dose-finding study of the pan-ErbB receptor tyrosine-kinase inhibitor CI-1033 in patients with pretreated metastatic breast cancer,’ *Cancer ChemotherPharmacol*.; 64(6):1139-48. doi: 10.1007/s00280-009-0975-z. Epub 2009 Mar 18. PMID: 19294387 |
| 50 | NCT00577122 | Medroxyprogesterone AND Cyclophosphamide AND Methotrexate AND Breast Cancer AND Miller K | Miller, K.D. et al. (2014) ‘A phase II study of medroxyprogesterone acetate in patients with hormone receptor negative metastatic breast cancer: translational breast cancer research consortium trial 007,’ *Breast Cancer Res Treat*.; 148(1):99-106. doi: 10.1007/s10549-014-3131-3. Epub 2014 Sep 26. PMID: 25257727 |
| 51 | NCT00017394 | Bevacizumab AND Vinorelbine AND Breast Cancer AND Burstein H | Burstein, H.J. et al. (2008) ‘VEGF as a marker for outcome among advanced breast cancer patients receiving anti-VEGF therapy with bevacizumab and vinorelbine chemotherapy,’ *Clin Cancer Res*.; 14(23):7871-7. doi: 10.1158/1078-0432.CCR-08-0593. PMID: 19047116 |
| 52 | NCT00736970 | Link | Seiler, M. et al. (2015) ‘Oral ridaforolimus plus trastuzumab for patients with HER2+ trastuzumab-refractory metastatic breast cancer,’ *Clin Breast Cancer*.; 15(1):60-5. doi: 10.1016/j.clbc.2014.07.008. Epub 2014 Aug 17. PMID: 25239224 |
| 53 | NCT00297596 | Link | Yardley, D.A. et al. (2010) ‘A phase II trial of oxaliplatin and trastuzumab in the treatment of HER2-positive metastatic breast cancer,’ *Cancer Invest*.; 28(8):865-71. doi: 10.3109/07357901003631031. PMID: 20690802 |
| 54 | NCT00172029 | Radiotherapy AND Reduced Dose AND Bone Metastases AND Breast Cancer AND Zoledronic Acid | Atahan, L. et al. (2010) ‘Zoledronic acid concurrent with either high- or reduced-dose palliative radiotherapy in the management of the breast cancer patients with bone metastases: a phase IV randomized clinical study,’ *Support Care Cancer*.; 18(6):691-8. doi: 10.1007/s00520-009-0663-x. Epub 2009 May 31. PMID: 19484483 |
| 55 | NCT00301899 | Link | Baselga, J. et al. (2010) ‘Phase II trial of pertuzumab and trastuzumab in patients with human epidermal growth factor receptor 2-positive metastatic breast cancer that progressed during prior trastuzumab therapy,’ *J ClinOncol*.; 28(7):1138-44. doi: 10.1200/JCO.2009.24.2024. Epub 2010 Feb 1. PMID: 20124182 |
| 56 | NCT00213980 | Bone Mineral Density AND Zoledronate AND Breast Cancer AND Mulkerin D | Leal, T. et al. (2010) ‘Randomized trial of adjuvant zoledronic acid in postmenopausal women with high-risk breast cancer,’ *Clin Breast Cancer*.; 10(6):471-6. doi: 10.3816/CBC.2010.n.062. PMID: 21147691 |
| 57 | NCT00976365 | THL-P AND Breast Cancer AND Chang K | Kuo, W.H., Yao, C.A., Lin, C.H. & Chang, K.J. (2012) ‘Safety and Efficacy of Tien-Hsien Liquid Practical in Patients with Refractory Metastatic Breast Cancer: A Randomized, Double-Blind, Placebo-Controlled, Parallel-Group, Phase IIa Trial,’ *Evid Based Complement Alternat Med*.; 2012:803239. doi: 10.1155/2012/803239. Epub 2012 Apr 1. PMID: 22548120 |
| 58 | NCT00508352 | Link | Caudrelier, J.M. et al. (2014) ‘IMRT sparing of normal tissues in locoregional treatment of breast cancer,’ *RadiatOncol*.; 9:161. doi: 10.1186/1748-717X-9-161. PMID: 25052720 |
| 59 | NCT00633750 | Erlotinib AND Breast Cancer AND Surgery AND Arteaga CL | Guix, M. et al. (2008) ‘Short preoperative treatment with erlotinib inhibits tumor cell proliferation in hormone receptor-positive breast cancers,’ *J ClinOncol*.; 26(6):897-906. doi: 10.1200/JCO.2007.13.5939. Epub 2008 Jan 7. PMID: 18180460 |
| 60 | NCT00148707 | Xyotax (CT-2103) AND Breast Cancer AND Shulman LN | Lin, N.U. et al. (2007) ‘Phase II study of CT-2103 as first- or second-line chemotherapy in patients with metastatic breast cancer: unexpected incidence of hypersensitivity reactions,’ *Invest New Drugs*.; 25(4):369-75. Epub 2007 Mar 8. PMID: 17345004 |
| 61 | NCT00245050 | Pyridoxine AND Hand-Foot Syndrome AND Doxorubicin AND Breast Cancer AND von Gruenigen V | vonGruenigen, V. et al. (2010) ‘A double-blind, randomized trial of pyridoxine versus placebo for the prevention of pegylated liposomal doxorubicin-related hand-foot syndrome in gynecologic oncology patients,’ *Cancer.*; 116(20):4735-43. doi: 10.1002/cncr.25262. PMID: 20629022 |
| 62 | NCT00107276 | Cyclophosphamide AND CapecitabineAND Breast Cancer AND Schott AF AND Albain KS | Schott, A.F. et al. (2012) ‘Phase II trial of simple oral therapy with capecitabine and cyclophosphamide in patients with metastatic breast cancer: SWOG S0430,’ *Oncologist*.; 17(2):179-87. doi: 10.1634/theoncologist.2011-0235. Epub 2012 Jan 20. PMID: 22267853 |
| 63 | NCT00540358 | Link | O'Shaughnessy, J. et al. (2011) ‘Iniparib plus chemotherapy in metastatic triple-negative breast cancer,’ *N Engl J Med*.; 364(3):205-14. doi: 10.1056/NEJMoa1011418. Epub 2011 Jan 5. PMID: 21208101 |
| 64 | NCT00156312 | Thymidine Phosphorylase AND Breast Cancer AND Docetaxel and Capecitabine AND Schott A | Layman, R.M. et al. (2007) ‘Neoadjuvant docetaxel and capecitabine and the use of thymidine phosphorylase as a predictive biomarker in breast cancer,’ *Clin Cancer Res*.; 13(14):4092-7. PMID: 17634534 |
| 65 | NCT00155259 | Docetaxel AND Cisplatin AND Capecitabine AND Breast Cancer AND Lu YS | Lu, Y.S. et al. (2011) ‘Phase II study of docetaxel, capecitabine, and cisplatin as neoadjuvant chemotherapy for locally advanced breast cancer,’ *Cancer ChemotherPharmacol*.; 67(6):1257-63. doi: 10.1007/s00280-010-1401-2. Epub 2010 Aug 11. PMID: 20700740 |
| 66 | NCT00107263 | Link | Hines, S.L. et al. (2009) ‘Immediate versus delayed zoledronic acid for prevention of bone loss in postmenopausal women with breast cancer starting letrozole after tamoxifen-N03CC,’ *Breast Cancer Res Treat*.; 117(3):603-9. doi: 10.1007/s10549-009-0332-2. Epub 2009 Feb 12. PMID: 19214743 |
| 67 | NCT00083993 | Link | Wolff, A.C. et al. (2013) ‘Randomized phase III placebo-controlled trial of letrozole plus oral temsirolimus as first-line endocrine therapy in postmenopausal women with locally advanced or metastatic breast cancer,’ *J ClinOncol*.; 31(2):195-202. doi: 10.1200/JCO.2011.38.3331. Epub 2012 Dec 10. PMID: 23233719 |
| 68 | NCT00273208 | Fatigue AND Breast Cancer Survivors AND Helzlsouer KJ | Appling, S.E. et al. (2012) ‘Fatigue in breast cancer survivors: the impact of a mind-body medicine intervention,’ *OncolNurs Forum*.; 39(3):278-86. doi: 10.1188/12.ONF.278-286. PMID: 22543386 |
| 69 | NCT00903656 | Lapatinib AND Caelyx AND Breast Cancer AND Trastuzumab AND Lang A | Pircher, M. et al. (2015) ‘Lapatinib-plus-pegylated liposomal doxorubicin in advanced HER2-positive breast cancer following trastuzumab: a phase II trial,’ *Anticancer Res*.; 35(1):517-21. PMID: 25550597 |
| 70 | NCT00008359 | Link | Walsh, T.J. et al. (2004) ‘Caspofungin versus liposomal amphotericin B for empirical antifungal therapy in patients with persistent fever and neutropenia,’ *N Engl J Med*.; 351(14):1391-402. PMID: 15459300 |
| 71 | NCT00092183 | Link | Warr, D.G. et al. (2005) ‘Efficacy and tolerability of aprepitant for the prevention of chemotherapy-induced nausea and vomiting in patients with breast cancer after moderately emetogenic chemotherapy,’ *J ClinOncol*.; 23(12):2822-30. PMID: 15837996 |
| 72 | NCT00031876 | Link | Uhlmann, C. et al. (2004) ‘Capecitabine with weekly paclitaxel for advanced breast cancer: a phase I dose-finding trial, Oncology’ 67(2):117-22. PMID: 15539915 |
| 73 | NCT00028535 | Interleukin-12 AND Paclitaxel AND Trastuzumab AND Cancer AND Carson W | Bekaii-Saab, T.S. et al. (2009) ‘A phase I trial of paclitaxel and trastuzumab in combination with interleukin-12 in patients with HER2/neu-expressing malignancies,’ *Mol Cancer Ther*.; 8(11):2983-91. doi: 10.1158/1535-7163.MCT-09-0820. Epub 2009 Nov 3. PMID: 19887543 |
| 74 | NCT00104702 | Radiotherapy AND Surgery AND Breast Cancer AND Hannoun-Levi AND Women | Hannoun-Levi, J.M. et al. (2013) ‘GERICO-03 phase II trial of accelerated and partial breast irradiation in elderly women: feasibility, reproducibility, and impact on functional status, Brachytherapy’ 12(4):285-92. doi: 10.1016/j.brachy.2012.06.004. Epub 2013 Feb 12. PMID: 23415050 |
| 75 | NCT00228358 | HER-2/Neu Specific T Cells AND Cancer AND Disis M | Disis, M.L. et al. (2014) ‘HER-2/neu vaccine-primed autologous T-cell infusions for the treatment of advanced stage HER-2/neu expressing cancers,’ *Cancer ImmunolImmunother*.; 63(2):101-9. doi: 10.1007/s00262-013-1489-4. Epub 2013 Oct 26. PMID: 24162107 |
| 76 | NCT00381901 | Link | Pivot, X. et al. (2013) ‘6 months versus 12 months of adjuvant trastuzumab for patients with HER2-positive early breast cancer (PHARE): a randomised phase 3 trial,’ *Lancet Oncol*.; 14(8):741-8. doi: 10.1016/S1470-2045(13)70225-0. Epub 2013 Jun 11. PMID: 23764181 |
| 77 | NCT00119262 | Bevacizumab AND Doxorubicin AND Cyclophosphamide AND Paclitaxel AND Lymph Node Positive Breast Cancer | Miller, K.D. et al. (2012) ‘A phase II pilot trial incorporating bevacizumab into dose-dense doxorubicin and cyclophosphamide followed by paclitaxel in patients with lymph node positive breast cancer: a trial coordinated by the Eastern Cooperative Oncology Group,’ *Ann Oncol*.; 23(2):331-7. doi: 10.1093/annonc/mdr344. Epub 2011 Aug 4. PMID: 21821545 |
| 78 | NCT00098605 | Lapatinib AND Brain Metastases AND Breast Cancer AND Winer E | Lin, N.U. et al. (2008) ‘Phase II trial of lapatinib for brain metastases in patients with human epidermal growth factor receptor 2-positive breast cancer,’ *J ClinOncol*.; 26(12):1993-9. doi: 10.1200/JCO.2007.12.3588. PMID: 18421051 |
| 79 | NCT00691678 | Glucosamine AND Chondroitin AND Aromatase Inhibitor Induced Joint Symptoms AND Breast Cancer AND Hershman D | Greenlee, H. et al. (2013) ‘Phase II study of glucosamine with chondroitin on aromatase inhibitor-associated joint symptoms in women with breast cancer,’ *Support Care Cancer*.; 21(4):1077-87. doi: 10.1007/s00520-012-1628-z. Epub 2012 Nov 1. PMID: 23111941 |
| 80 | NCT00721565 | Link | Rogers, L.Q. et al. (2009) ‘Physical activity and health outcomes three months after completing a physical activity behavior change intervention: persistent and delayed effects,’ *Cancer Epidemiol Biomarkers Prev*.; 18(5):1410-8. doi: 10.1158/1055-9965.EPI-08-1045. Epub 2009 Apr 21. PMID: 19383889 |
| 81 | NCT00583726 | Link | Djuric, Z. et al. (2012) ‘Lifestyle factors associated with serum N-3 fatty acid levels in breast cancer patients,’ *Breast*.; 21(4):608-11. doi: 10.1016/j.breast.2012.02.003. Epub 2012 Feb 28. PMID: 22377591 |
| 82 | NCT00107510 | Link | Roy, V. et al. (2013) ‘A Phase II trial of docetaxel and carboplatin administered every 2 weeks as preoperative therapy for stage II or III breast cancer: NCCTG study N0338,’ *Am J ClinOncol*.; 36(6):540-4. doi: 10.1097/COC.0b013e318256f619. PMID: 22868240 |
| 83 | NCT00126451 | Link | Vansteenkiste, J. et al. (2008) ‘Early phase II trial of oral vorinostat in relapsed or refractory breast, colorectal, or non-small cell lung cancer,’ *Invest New Drugs*.; 26(5):483-8. doi: 10.1007/s10637-008-9131-6. Epub 2008 Apr 19. PMID: 18425418 |
| 84 | NCT00258960 | Caelyx, Cyclophosphamide and Herceptin in Patients With Metastatic Breast Cancer AND Martín M | Martin, M. et al. (2011) ‘Pegylated liposomal doxorubicin in combination with cyclophosphamide and trastuzumab in HER2-positive metastatic breast cancer patients: efficacy and cardiac safety from the GEICAM/2004-05 study,’ *Ann Oncol*.; 22(12):2591-6. doi: 10.1093/annonc/mdr024. Epub 2011 Mar 17. PMID: 21421542 |
| 85 | NCT00288444 | Docetaxel AND Lonafarnib AND Cancer AND Kauh J | Kauh, J. et al. (2011) ‘Farnesyl transferase expression determines clinical response to the docetaxel-lonafarnib combination in patients with advanced malignancies,’ *Cancer*; 117(17):4049-59. doi: 10.1002/cncr.26004. Epub 2011 Mar 1. PMID: 21365629 |
| 86 | NCT01880580 | Link | O'Connell, A.M. &Kawakyu-O'Connor, D. (2012) ‘Dedicated Cone-beam Breast Computed Tomography and Diagnostic Mammography: Comparison of Radiation Dose, Patient Comfort, And Qualitative Review of Imaging Findings in BI-RADS 4 and 5 Lesions,’ *J Clin Imaging Sci*.; 2:7. doi: 10.4103/2156-7514.93274. Epub 2012 Feb 25. PMID: 22439131 |
| 87 | NCT02056067 | Link | Irwin, M.L. et al. (2015) ‘Randomized exercise trial of aromatase inhibitor-induced arthralgia in breast cancer survivors,’ *J ClinOncol*.; 33(10):1104-11. doi: 10.1200/JCO.2014.57.1547. Epub 2014 Dec 1. PMID: 25452437 |
| 88 | NCT00548899 | Link | Loibl, S. et al. (2014) ‘Sorafenib in the Treatment of Early Breast Cancer: Results of the Neoadjuvant Phase II Study – SOFIA,’ *Breast Care* (Basel); 9(3):169-74. doi: 10.1159/000363430. PMID: 25177258 |
| 89 | NCT00092196 | Link | Warr, D.G. et al. (2005) ‘Efficacy and tolerability of aprepitant for the prevention of chemotherapy-induced nausea and vomiting in patients with breast cancer after moderately emetogenic chemotherapy,’ *J ClinOncol*.; 23(12):2822-30. PMID: 15837996 |
| 90 | NCT00347438 | Capecitabine AND Breast Cancer AND Olopade O | Arowolo, O.A. (2013) ‘Neo-adjuvant capecitabine chemotherapy in women with newly diagnosed locally advanced breast cancer in a resource-poor setting (Nigeria): efficacy and safety in a phase II feasibility study,’ *Breast J*.; 19(5):470-7. doi: 10.1111/tbj.12149. Epub 2013 Jul 19. PMID: 23865786 |
| 91 | NCT00369655 | VEGF Trap AND Breast Cancer AND Anthracycline AND Taxane AND Perez E | Sideras, K. et al. (2012) ‘North central cancer treatment group (NCCTG) N0537: phase II trial of VEGF-trap in patients with metastatic breast cancer previously treated with an anthracycline and/or a taxane,’ *Clin Breast Cancer*.; 12(6):387-91. doi: 10.1016/j.clbc.2012.09.007. Epub 2012 Oct 17. PMID: 23083501 |
| 92 | NCT01525264 | Breast Cancer Screening Intervention, Korean Immigrants & Mammography AND Lee E | Lee, E. et al. (2014) ‘The effect of a couples intervention to increase breast cancer screening among koreanamericans,’ *OncolNurs Forum*; 41(3):E185-93. doi: 10.1188/14.ONF.E185-E193. PMID: 24769601 |
| 93 | NCT01395459 | Link | Emmons, K.M. et al. (2011) ‘Prevalence and implications of multiple cancer screening needs among Hispanic community health center patients,’ *Cancer Causes Control*; 22(9):1343-9. doi: 10.1007/s10552-011-9807-7. Epub 2011 Jul 5. PMID: 21728056 |
| 94 | NCT00079170 | Docetaxel AND Garlic AND Breast Cancer AND Cox M | Cox, M.C. et al. (2006) ‘Influence of garlic (Allium sativum) on the pharmacokinetics of docetaxel,’ *Clin Cancer Res*.; 12(15):4636-40. PMID: 16899612 |
| 95 | NCT00096109 | Tanespimycin AND Breast Cancer AND Gartner E | Gartner, E.M. et al. (2012) ‘A phase II study of 17-allylamino-17-demethoxygeldanamycin in metastatic or locally advanced, unresectable breast cancer,’ *Breast Cancer Res Treat*.; 131(3):933-7. doi: 10.1007/s10549-011-1866-7. Epub 2011 Nov 15. PMID: 22083229 |
| 96 | NCT00166543 | TAS-108 AND Breast Cancer AND Ingle J | Buzdar, A. et al. (2012) ‘Randomized double-blind phase 2 trial of 3 doses of TAS-108 in patients with advanced or metastatic postmenopausal breast cancer,’ *Cancer*; 118(13):3244-53. doi: 10.1002/cncr.26419. Epub 2011 Nov 1. PMID: 22045595 |
| 97 | NCT00291473 | CHP-HER2 AND CHP-NY-ESO-1 AND Cancer AND Shiku H | Aoki, M. et al. (2009) ‘Antibody responses against NY-ESO-1 and HER2 antigens in patients vaccinated with combinations of cholesteryl pullulan (CHP)-NY-ESO-1 and CHP-HER2 with OK-432,’ *Vaccine*; 27(49):6854-61. doi: 10.1016/j.vaccine.2009.09.018. Epub 2009 Sep 15. PMID: 19761832 |
| 98 | NCT00534417 | Capecitabine AND Fulvestrant AND Breast Cancer AND Schwartzberg L | Schwartzberg, L.S. et al. (2014) ‘Phase II trial of fulvestrant with metronomic capecitabine for postmenopausal women with hormone receptor-positive, HER2-negative metastatic breast cancer,’ *Clin Breast Cancer*; 14(1):13-9. doi: 10.1016/j.clbc.2013.09.003. Epub 2013 Sep 27. PMID: 24268206 |
| 99 | NCT01206881 | Link | Tuxen, M.K. et al. (2014) ‘Phase II study of neoadjuvant pegylated liposomal doxorubicin and cyclophosphamide ± trastuzumab followed by docetaxel in locally advanced breast cancer,’ *ActaOncol*.; 53(10):1440-5. doi: 10.3109/0284186X.2014.921727. Epub 2014 Jul 3. PMID: 24991893 |
| 100 | NCT00932373 | Link | Beeram, M. et al. (2012) ‘A phase 1 study of weekly dosing of trastuzumabemtansine (T-DM1) in patients with advanced human epidermal growth factor 2-positive breast cancer,’ *Cancer*; 118(23):5733-40. doi: 10.1002/cncr.27622. Epub 2012 May 30. PMID: 22648179 |
| 101 | NCT00089661 | Link | Ellis, G.K. et al. (2008) ‘Randomized trial of denosumab in patients receiving adjuvant aromatase inhibitors for nonmetastatic breast cancer,’ *J ClinOncol*.; 26(30):4875-82. doi: 10.1200/JCO.2008.16.3832. Epub 2008 Aug 25. PMID: 18725648 |
| 102 | NCT00623831 | Mixed Bacteria Vaccine (MBV) AND Tumors Expressing NY-ESO-1 Antigen AND Jaeger E | Karbach, J. et al. (2012) ‘Phase I clinical trial of mixed bacterial vaccine (Coley's toxins) in patients with NY-ESO-1 expressing cancers: immunological effects and clinical activity,’ *Clin Cancer Res*.; 18(19):5449-59. doi: 10.1158/1078-0432.CCR-12-1116. Epub 2012 Jul 30. PMID: 22847809 |
| 103 | NCT00090844 | Triptorelin AND Ovarian Function AND Chemotherapy AND Breast Cancer AND Munster P | Munster, P.N. et al. (2012) ‘Randomized trial using gonadotropin-releasing hormone agonist triptorelin for the preservation of ovarian function during (neo)adjuvant chemotherapy for breast cancer,’ *J ClinOncol*.; 30(5):533-8. doi: 10.1200/JCO.2011.34.6890. Epub 2012 Jan 9. PMID: 22231041 |
| 104 | NCT00641628 | Link | Paepke, S. et al. (2009) ‘Subcutaneous mastectomy with conservation of the nipple-areola skin: broadening the indications,’ *Ann Surg*.; 250(2):288-92. doi: 10.1097/SLA.0b013e3181b0c7d8. PMID: 19638905 |
| 105 | NCT00728442 | Impact of OncoDoc2 on Guideline Compliance in the Management of Breast Cancer | Seroussi, B. et al. (2013) ‘Which patients may benefit from the use of a decision support system to improve compliance of physician decisions with clinical practice guidelines: a case study with breast cancer involving data mining,’ *Stud Health Technol Inform*.; 192:534-8. PMID: 23920612 |
| 106 | NCT00572598 | Link | Kurdziel, K.A. et al. (2011) ‘Human dosimetry and preliminary tumor distribution of 18F-fluoropaclitaxel in healthy volunteers and newly diagnosed breast cancer patients using PET/CT,’ *J Nucl Med*.; 52(9):1339-45. doi: 10.2967/jnumed.111.091587. Epub 2011 Aug 17. PMID: 21849404 |
| 107 | NCT00477919 | Link | Blum, D. et al. (2012) ‘Electronic monitoring of symptoms and syndromes associated with cancer: methods of a randomized controlled trial SAKK 95/06 E-MOSAIC,’ *BMC Palliat Care*.; 11:19. doi: 10.1186/1472-684X-11-19. PMID: 23006802 |
| 108 | NCT00429247 | Trastuzumab AND Breast Cancer AND Georgoulias V AND Observation | Georgoulias, V. et al. (2012) ‘Trastuzumab decreases the incidence of clinical relapses in patients with early breast cancer presenting chemotherapy-resistant CK-19mRNA-positive circulating tumor cells: results of a randomized phase II study,’ *Ann Oncol*.; 23(7):1744-50. doi: 10.1093/annonc/mds020. Epub 2012 Feb 29. PMID: 22377561 |
| 109 | NCT00023790 | Photodynamic Therapy AND Skin Cancer or Solid Tumors AND Remick S AND PC 4-PDT | Kinsella, T.J. et al. (2011) ‘Preliminary clinical and pharmacologic investigation of photodynamic therapy with the silicon phthalocyanine photosensitizer pc 4 for primary or metastatic cutaneous cancers,’ *Front Oncol*.; 1:14. doi: 10.3389/fonc.2011.00014. eCollection 2011. PMID: 22649754 |
| 110 | NCT00699101 | Link | Cuttino, L.W. et al. (2014) ‘Long-term results from the Conturamultilumen balloon breast brachytherapy catheter phase 4 registry trial,’ *Int J RadiatOncolBiol Phys*.; 90(5):1025-9. doi: 10.1016/j.ijrobp.2014.08.341. Epub 2014 Oct 13. PMID: 25442036 |
| 111 | NCT00952692 | Link | Hamilton, E. et al. (2012) ‘Phase 1 clinical trial of HER2-specific immunotherapy with concomitant HER2 kinase inhibition [corrected],’ *J Transl Med*.; 10:28. doi: 10.1186/1479-5876-10-28. PMID: 22325452 |
| 112 | NCT00284336 | Caelyx AND Elderly AND Breast Cancer AND Wildiers H | Jurcut, R. (2008) ‘Strain rate imaging detects early cardiac effects of pegylated liposomal Doxorubicin as adjuvant therapy in elderly patients with breast cancer,’ *J Am SocEchocardiogr*.; 21(12):1283-9. doi: 10.1016/j.echo.2008.10.005. PMID: 19041569 |
| 113 | NCT00319254 | SKI-606 (Bosutinib) AND  Advanced (Metastatic) Breast Cancer | Campone, M. et al. (2012) ‘Phase II study of single-agent bosutinib, a Src/Abl tyrosine kinase inhibitor, in patients with locally advanced or metastatic breast cancer pretreated with chemotherapy,’ *Ann Oncol*.; 23(3):610-7. doi: 10.1093/annonc/mdr261. Epub 2011 Jun 23. PMID: 21700731 |
| 114 | NCT00386087 | Enzastaurin AND Breast Cancer AND Anthracycline AND Taxane | Mina, L. et al. (2009) ‘A phase II study of oral enzastaurin in patients with metastatic breast cancer previously treated with an anthracycline and a taxane containing regimen,’ *Invest New Drugs*.; 27(6):565-70. doi: 10.1007/s10637-009-9220-1. Epub 2009 Feb 13. PMID: 19214387 |
| 115 | NCT00263211 | Platelet Function AND Circulating Cancer Cells AND Breast Cancer AND Weilbaecher K | Roop, R.P. et al. (2013) ‘A randomized phase II trial investigating the effect of platelet function inhibition on circulating tumor cells in patients with metastatic breast cancer,’ *Clin Breast Cancer*.; 13(6):409-15. doi: 10.1016/j.clbc.2013.08.006. PMID: 24267729 |
| 116 | NCT00291109 | Letrozole AND Fabian CJ AND Breast Cancer | Frank, D.H. et al. (2009) ‘Digital image analysis of breast epithelial cells collected by random periareolar fine-needle aspirates (RPFNA) from women at high risk for breast cancer taking hormone replacement and the aromatase inhibitor, letrozole, for six months,’ *Breast Cancer Res Treat*.; 115(3):661-8. doi: 10.1007/s10549-008-0274-0. PMID: 19125322 Epub 2009 Jan 6. |
| 117 | NCT00080665 | Link | Connolly, R.M. et al. (2011) ‘Docetaxel metabolism is not altered by imatinib: findings from an early phase study in metastatic breast cancer,’ *Breast Cancer Res Treat*.; 127(1):153-62. doi: 10.1007/s10549-011-1413-6. Epub 2011 Feb 25. PMID: 21350820 |
| 118 | NCT00237627 | Link | Dees, E.C. et al. (2008) ‘A phase I and pharmacologic study of the combination of bortezomib and pegylated liposomal doxorubicin in patients with refractory solid tumors,’ *Cancer ChemotherPharmacol*.; 63(1):99-107. doi: 10.1007/s00280-008-0716-8. Epub 2008 Mar 8. PMID: 18327587 |
| 119 | NCT00026117 | Link | Loprinzi, C.L. et al. (2005) ‘Evaluation of shark cartilage in patients with advanced cancer: a North Central Cancer Treatment Group trial,’ *Cancer*; 104(1):176-82. PMID: 15912493 |
| 120 | NCT00572416 | Link | Berger, A.M. et al. (2007) ‘Values of sleep/wake, activity/rest, circadian rhythms, and fatigue prior to adjuvant breast cancer chemotherapy,’ *J Pain Symptom Manage*.; 33(4):398-409. PMID: 17397701 |
| 121 | NCT00386217 | Psychosocial Impact of Cancer-Related Female Infertility AND Schover LR | Canada, A.L. &Schover, L.R. (2012) ‘The psychosocial impact of interrupted childbearing in long-term female cancer survivors,’ *Psychooncology*; 21(2):134-43. doi: 10.1002/pon.1875. Epub 2010 Dec 2. PMID: 22271533 |
| 122 | NCT00028405 | Photoactivation AND Solid Tumors | Lustig, R.A. (2003) ‘A multicenter Phase I safety study of intratumoralphotoactivation of talaporfin sodium in patients with refractory solid tumors,’ *Cancer*; 98(8):1767-71. PMID: 14534895 |
| 123 | NCT01158274 | RO4929097 AND Capecitabine AND Solid Tumors AND LoConte N | LoConte, N.K. et al. (2015) ‘A multicenter phase 1 study of γ -secretase inhibitor RO4929097 in combination with capecitabine in refractory solid tumors,’ *Invest New Drugs*.; 33(1):169-76. doi: 10.1007/s10637-014-0166-6. Epub 2014 Oct 17. PMID: 25318436 |
| 124 | NCT00256698 | Anastrozole AND Fulvestrant AND Henriksson R | Bergh, J. et al. (2012) ‘FACT: an open-label randomized phase III study of fulvestrant and anastrozole in combination compared with anastrozole alone as first-line therapy for patients with receptor-positive postmenopausal breast cancer,’ *J ClinOncol*.; 30(16):1919-25. doi: 10.1200/JCO.2011.38.1095. Epub 2012 Feb 27. PMID: 22370325 |
| 125 | NCT00857012 | Link | Hadji, P. et al. (2014) ‘COMPliance and Arthralgia in Clinical Therapy: the COMPACT trial, assessing the incidence of arthralgia, and compliance within the first year of adjuvant anastrozole therapy,’ *Ann Oncol*.; 25(2):372-7. doi: 10.1093/annonc/mdt513. Epub 2013 Dec 18. PMID: 24355487 |
| 126 | NCT00388115 | Radiofrequency Ablation AND Breast Cancer AND Khatri V | Khatri, V.P. et al. (2007) ‘A phase II trial of image-guided radiofrequency ablation of small invasive breast carcinomas: use of saline-cooled tip electrode,’ *Ann SurgOncol*.; 14(5):1644-52. Epub 2007 Feb 15. PMID: 17508251 |
| 127 | NCT00191347 | Pemetrexed and Gemcitabine in Patients With Metastatic Breast Cancer | Dent, S.F. et al. (2010) ‘A phase II study of biweekly pemetrexed and gemcitabine in patients with metastatic breast cancer,’ *Cancer ChemotherPharmacol*.; 65(3):557-61. doi: 10.1007/s00280-009-1064-z. Epub 2009 Jul 11. PMID: 19593565 |
| 128 | NCT00911911 | Proteomic Signature AND Breast Cancer AND Tumor Response AND Chemotherapy AND BONNETERRE J | Bonneterre, J. et al. (2013) ‘Plasma and tissue proteomic prognostic factors of response in primary breast cancer patients receiving neoadjuvant chemotherapy,’ *Oncol Rep*.; 29(1):355-61. doi: 10.3892/or.2012.2090. Epub 2012 Oct 19. PMID: 23117275 |
| 129 | NCT00324259 | Link | Ellis, M.J. et al. (2009) ‘Lower-dose vs high-dose oral estradiol therapy of hormone receptor-positive, aromatase inhibitor-resistant advanced breast cancer: a phase 2 randomized study,’ *JAMA*; 302(7):774-80. doi: 10.1001/jama.2009.1204. PMID: 19690310 |
| 130 | NCT00030537 | Link | Tan, A.R. et al. (2004) ‘Evaluation of biologic end points and pharmacokinetics in patients with metastatic breast cancer after treatment with erlotinib, an epidermal growth factor receptor tyrosine kinase inhibitor,’ *J ClinOncol*.; 22(15):3080-90. PMID: 15284258 |
| 131 | NCT01424956 | Breast Ultrasound AND Screening Mammogram AND Giger M | Drukker, K., Horsch, K.J., Pesce, L.L. & Giger, M.L. (2013) ‘Interreader scoring variability in an observer study using dual-modality imaging for breast cancer detection in women with dense breasts,’ *AcadRadiol*.; 20(7):847-53. doi: 10.1016/j.acra.2013.02.007. Epub 2013 Apr 17. PMID: 23601952 |
| 132 | NCT00393939 | Docetaxel In Combination With Sunitinib Versus Docetaxel In The First-Line Treatment Of Advanced Breast Cancer | Bergh, J. et al. (2012) ‘First-line treatment of advanced breast cancer with sunitinib in combination with docetaxel versus docetaxel alone: results of a prospective, randomized phase III study,’ *J ClinOncol*.; 30(9):921-9. doi: 10.1200/JCO.2011.35.7376. Epub 2012 Feb 13. PMID: 22331954 |
| 133 | NCT00365417 | Bevacizumab AND Doxorubicin AND Cyclophosphamide AND Docetaxel AND Capecitabine AND Wolmark N | Rastogi, P. et al. (2011) ‘Concurrent bevacizumab with a sequential regimen of doxorubicin and cyclophosphamide followed by docetaxel and capecitabine as neoadjuvant therapy for HER2- locally advanced breast cancer: a phase II trial of the NSABP Foundation Research Group,’ *Clin Breast Cancer*.; 11(4):228-34. doi: 10.1016/j.clbc.2011.04.001. Epub 2011 May 4. PMID: 21684812 |
| 134 | NCT00606931 | Link | Kalinyak, J.E. et al. (2011) ‘PET-guided breast biopsy,’ *Breast J*.; 17(2):143-51. doi: 10.1111/j.1524-4741.2010.01044.x. Epub 2011 Jan 31. PMID: 21276128 |
| 135 | NCT00191269 | Gemcitabine Monotherapy AND Anthracycline AND Taxane AND Breast Cancer | Suzuki, Y. et al. (2009) ‘Phase II study of gemcitabine monotherapy as a salvage treatment for Japanese metastatic breast cancer patients after anthracycline and taxane treatment,’ *Jpn J ClinOncol*.; 39(11):699-706. doi: 10.1093/jjco/hyp103. Epub 2009 Sep 22. PMID: 19776022 |
| 136 | NCT00005980 | Link | Coleman, R.E. et al. (2006) ‘A randomised phase II study of two different schedules of pegylated liposomal doxorubicin in metastatic breast cancer (EORTC-10993),’ Eur J Cancer; 42(7):882-7. Epub 2006 Mar 7. PMID: 16520033 |
| 137 | NCT00012025 | Link | Ingle, J.N. et al. (2006) ‘Fulvestrant in women with advanced breast cancer after progression on prior aromatase inhibitor therapy: North Central Cancer Treatment Group Trial N0032,’ *J ClinOncol*.; 24(7):1052-6. PMID: 16505423 |
| 138 | NCT00054418 | Link | Hines, S.L. et al. (2009) ‘Phase III randomized, placebo-controlled, double-blind trial of risedronate for the prevention of bone loss in premenopausal women undergoing chemotherapy for primary breast cancer,’ *J ClinOncol*.; 27(7):1047-53. doi: 10.1200/JCO.2008.19.1783. Epub 2008 Dec 15. PMID: 19075260 |
| 139 | NCT02254005 | Bivatuzumab AND CD44v6 AND Breast Cancer | Rupp, U. et al. (2007) ‘Safety and pharmacokinetics of bivatuzumabmertansine in patients with CD44v6-positive metastatic breast cancer: final results of a phase I study,’ *Anticancer Drugs*; 18(4):477-85. PMID: 17351401 |
| 140 | NCT00089141 | Link | Martin, P.J. et al. (2009) ‘Evaluation of mycophenolate mofetil for initial treatment of chronic graft-versus-host disease,’ *Blood*; 113(21):5074-82. doi: 10.1182/blood-2009-02-202937. Epub 2009 Mar 6. PMID: 19270260 |
| 141 | NCT00062400 | Attitudes AND Risk AND Infertility AND Breast Cancer AND Neskovic-Konstantinovic Z AND EORTC study 10002 BIG 3-98 | Senkus, e. et al. (2014) ‘Attitudes of young patients with breast cancer toward fertility loss related to adjuvant systemic therapies. EORTC study 10002 BIG 3-98,’ *Psychooncology*; 23(2):173-82. doi: 10.1002/pon.3384. Epub 2013 Aug 29. PMID: 24038775 |
| 142 | NCT00020332 | Docetaxel AND Flavopiridol AND Breast Cancer AND Tan AR | Tan, A.R. et al. (2004) ‘Phase I trial of the cyclin-dependent kinase inhibitor flavopiridol in combination with docetaxel in patients with metastatic breast cancer,’ *Clin Cancer Res*.; 10(15):5038-47. PMID: 15297405 |
| 143 | NCT01784393 | Chemoradiation AND Capecitabine AND Pain AND Bone Metastasis AND Kundel Y AND Breast Cancer | Kundel, Y. et al. (2013) ‘Phase II study of concurrent capecitabine and external beam radiotherapy for pain control of bone metastases of breast cancer origin,’ *PLoS One*; 8(7):e68327. doi: 10.1371/journal.pone.0068327. Print 2013. PMID: 23874586 |
| 144 | NCT00041808 | Magnetic-Targeted AND Doxorubicin AND Cancer AND Liver AND Koda J | Wilson, M.W. et al. (2004) ‘Hepatocellular carcinoma: regional therapy with a magnetic targeted carrier bound to doxorubicin in a dual MR imaging/ conventional angiography suite--initial experience with four patients,’ *Radiology*; 230(1):287-93. PMID: 14695402 |
| 145 | NCT01738048 | Persistent Pain After Reconstruction Following Mastectomy AND Andersen KG | Kilt, A. et al. (2013) ‘Breast reconstruction with an expander prosthesis following mastectomy does not cause additional persistent pain: a nationwide cross-sectional study,’ *J PlastReconstrAesthet Surg*.; 66(12):1652-8. doi: 10.1016/j.bjps.2013.07.015. Epub 2013 Aug 2. PMID: 23911718 |
| 146 | NCT00127465 | Link | Jones, R.B. et al. (2006) ‘Effect of different forms of information produced for cancer patients on their use of the information, social support, and anxiety: randomised trial,’ *BMJ*; 332(7547):942-8. Epub 2006 Apr 5. PMID: 16597660 |
| 147 | NCT00085020 | GW572016 AND Trastuzumab AND Breast Cancer AND Pegram MD | Storniolo, A.M. et al. (2008) ‘Phase I dose escalation and pharmacokinetic study of lapatinib in combination with trastuzumab in patients with advanced ErbB2-positive breast cancer,’ *J ClinOncol*.; 26(20):3317-23. doi: 10.1200/JCO.2007.13.5202. Epub 2008 May 19. PMID: 18490651 |
| 148 | NCT00089999 | Link | Lipton, A. et al. (2011) ‘Human epidermal growth factor receptor 2 (HER2) extracellular domain levels are associated with progression-free survival in patients with HER2-positive metastatic breast cancer receiving lapatinib monotherapy,’ *Cancer*; 117(21):5013-20. doi: 10.1002/cncr.26101. Epub 2011 Mar 31. PMID: 21456017 |
| 149 | NCT00349011 | Counseling AND Menopausal AND Decision-Making AND Breast Cancer AND Matloff ET | Matloff, E.T. et al. (2006) ‘Healthy women with a family history of breast cancer: impact of a tailored genetic counseling intervention on risk perception, knowledge, and menopausal therapy decision making,’ *J Womens Health (Larchmt)*.; 15(7):843-56. PMID: 16999640 |
| 150 | NCT00526149 | Link | Schöffski, P. et al. (2010) ‘Multicentric parallel phase II trial of the polo-like kinase 1 inhibitor BI 2536 in patients with advanced head and neck cancer, breast cancer, ovarian cancer, soft tissue sarcoma and melanoma. The first protocol of the European Organization for Research and Treatment of Cancer (EORTC) Network Of Core Institutes (NOCI),’ *Eur J Cancer*; 46(12):2206-15. doi: 10.1016/j.ejca.2010.03.039. Epub 2010 May 13. PMID: 20471824 |
| 151 | NCT01157026 | Link | Nesaretnam, K. et al. (2010) ‘Effectiveness of tocotrienol-rich fraction combined with tamoxifen in the management of women with early breast cancer: a pilot clinical trial,’ *Breast Cancer Res*.; 12(5):R81. doi: 10.1186/bcr2726. Epub 2010 Oct 8. PMID: 20929592 |
| 152 | NCT00193908 | Link | Graham, P.H. et al. (2013) ‘A paired, double-blind, randomized comparison of a moisturizing durable barrier cream to 10% glycerine cream in the prophylactic management of postmastectomy irradiation skin care: trans Tasman Radiation Oncology Group (TROG) 04.01,’ *Int J RadiatOncolBiol Phys*.; 86(1):45-50. doi: 10.1016/j.ijrobp.2012.12.009. Epub 2013 Feb 12. PMID: 23414763 |
| 153 | NCT00401427 | Link | De Maio, E. et al. (2007) ‘Vinorelbine plus 3-weekly trastuzumab in metastatic breast cancer: a single-centre phase 2 trial,’ *BMC Cancer*; 7:50. PMID: 17374151 |
| 154 | NCT00462956 | GW572016 AND Metastatic Breast Cancer AND EGF100642 | Toi, M. et al. (2009) ‘Lapatinib monotherapy in patients with relapsed, advanced, or metastatic breast cancer: efficacy, safety, and biomarker results from Japanese patients phase II studies,’ *Br J Cancer*; 101(10):1676-82. doi: 10.1038/sj.bjc.6605343. Epub 2009 Oct 20. PMID: 19844234 |
| 155 | NCT00078000 | CTN | Keyvanjah, K. et al. (2012) ‘Soluble KIT correlates with clinical outcome in patients with metastatic breast cancer treated with sunitinib,’ *J Transl Med*.; 10:165. doi: 10.1186/1479-5876-10-165. PMID: 22897944 |
| 156 | NCT02005549 | Bevacizumab AND Docetaxel AND Capecitabine AND Breast Cancer AND ML19869 | Greil, R. et al. (2009) ‘Neoadjuvant bevacizumab, docetaxel and capecitabinecombination therapy for HER2/neu-negative invasive breast cancer: Efficacy and safety in a phase II pilot study,’ *Eur J SurgOncol.*; 35(10):1048-54. doi: 10.1016/j.ejso.2009.01.014. Epub 2009 Feb 27. PMID: 19250795 |
| 157 | NCT01110291 | Link | Hilli, J. et al. (2011) ‘NCT01110291: induction of CYP3A activity and lowered exposure to docetaxel in patients with primary breast cancer,’ *Cancer ChemotherPharmacol*.; 67(6):1353-62. doi: 10.1007/s00280-010-1426-6. Epub 2010 Aug 27. PMID: 20798939 |
| 158 | NCT00174434 | SU011248 AND Paclitaxel AND Breast Cancer AND First-Line Treatment | Kozloff, M. et al. (2010) ‘An exploratory study of sunitinib plus paclitaxel as first-line treatment for patients with advanced breast cancer,’ *Ann Oncol*.; 21(7):1436-41. doi: 10.1093/annonc/mdp565. Epub 2009 Dec 23. PMID: 20032126 |
| 159 | NCT00054145 | Link | Leighl, N.B. et al. (2008) ‘A Phase 2 study of perifosine in advanced or metastatic breast cancer,’ *Breast Cancer Res Treat*.; 108(1):87-92. Epub 2007 Apr 26. PMID: 17458693 |
| 160 | NCT00020579 | MS-275 AND Solid Tumors AND Lymphoma AND Kummar S | Kummar, S. et al. (2007) ‘Phase I trial of MS-275, a histone deacetylase inhibitor, administered weekly in refractory solid tumors and lymphoid malignancies,’ *Clin Cancer Res*.; 13(18 Pt 1):5411-7. PMID: 17875771 |
| 161 | NCT00299039 | Acetaminophen AND Ibuprofen AND Tylenol AND Breast AND Mitchell A | Mitchell, A., McCrea, P., Inglis, K. & Porter, G. (2012) ‘A randomized, controlled trial comparing acetaminophen plus ibuprofen versus acetaminophen plus codeine plus caffeine (Tylenol 3) after outpatient breast surgery,’ *Ann SurgOncol*.; 19(12):3792-800. doi: 10.1245/s10434-012-2447-7. Epub 2012 Jun 20. PMID: 22713999 |
| 162 | NCT00909649 | influence of fibrin glue on seroma formation after modified radical mastectomy AND El nakeeb A | El Nakeeb, A. (2009) ‘Influence of fibrin glue on seroma formation after modified radical mastectomy: a prospective randomized study,’ *Breast J*.; 15(6):671-2. doi: 10.1111/j.1524-4741.2009.00801.x. Epub 2009 Jun 24. PMID: 19558542 |
| 163 | NCT00024154 | Trastuzumab AND Gefitinib AND Breast Cancer AND Arteaga C | Arteaga, C.L. et al. (2008) ‘A phase I-II study of combined blockade of the ErbB receptor network with trastuzumab and gefitinib in patients with HER2 (ErbB2)-overexpressing metastatic breast cancer,’ *Clin Cancer Res*.; 14(19):6277-83. doi: 10.1158/1078-0432.CCR-08-0482. PMID: 18829509 |
| 164 | NCT00107393 | Link | Ishida, T. et al. (2009) ‘Phase II study of capecitabine and trastuzumab combination chemotherapy in patients with HER2 overexpressing metastatic breast cancers resistant to both anthracyclines and taxanes,’ *Cancer ChemotherPharmacol*.; 64(2):361-9. doi: 10.1007/s00280-008-0882-8. Epub 2008 Dec 12. PMID: 19082596 |
| 165 | NCT00194714 | Link | Disis, M.L. et al. (2009) ‘Concurrent trastuzumab and HER2/neu-specific vaccination in patients with metastatic breast cancer,’ *J ClinOncol*.; 27(28):4685-92. doi: 10.1200/JCO.2008.20.6789. Epub 2009 Aug 31. PMID: 19720923 |
| 166 | NCT00198250 | Link | Carpenter, J.S. et al. (2007) ‘Randomized, double-blind, placebo-controlled crossover trials of venlafaxine for hot flashes after breast cancer,’ *Oncologist*; 12(1):124-35. PMID: 17227907 |
| 167 | NCT00193076 | Link | Yardley, D.A. et al. (2008) ‘A phase II trial of gemcitabine/carboplatin with or without trastuzumab in the first-line treatment of patients with metastatic breast cancer,’ *Clin Breast Cancer*; 8(5):425-31. doi: 10.3816/CBC.2008.n.051. PMID: 18952556 |
| 168 | NCT00615316 | Guaraná ("PaulliniaCupana") AND Radiation AND Fatigue AND Breast Cancer AND Del Giglio A | da Costa Miranda, V. et al. (2009) ‘Effectiveness of guaraná (Paulliniacupana) for postradiation fatigue and depression: results of a pilot double-blind randomized study,’ *J Altern Complement Med*.; 15(4):431-3. doi: 10.1089/acm.2008.0324. PMID: 19388866 |
